# Supplementary material for: Stochastic gene expression in Arabidopsis thaliana
Source: Nat Commun. 2017 Dec 14;8:2132. doi: 10.1038/s41467-017-02285-7 (PMC5730595; doi:10.1038/s41467-017-02285-7)
Supplement: Supplementary file 1 — Supplementary Information [file 41467_2017_2285_MOESM1_ESM.pdf]

Supplementary Note 1: Non-stationary auto-correlation of the two-stage gene expression model in presence of extrinsic noise

In the experiments we can estimate the auto-correlation of the gene expression in Arabidopsis. To be able to compare the estimation with the result from the two-stage gene expression model, we need to calculate the non-stationary auto-correlation between protein abundance in case of extrinsic noise. At time $t_0 = 0$ KikG is converted from green to red, i.e., for $t > t_0$ one has a red and a green fluorescent protein population. The red protein population decays to zero due to protein degradation and the green population is building up from zero. We therefore consider a stochastic process with initial condition $n(t_0) = 0$. We denote the value at time t_2 conditioned on the value at time t_1 by: $n(t_2)|n(t_1)$ and the expectation value of n at time t_2 conditioned on the value $n(t_1)$ at time t_1 by $\langle n(t_2)|n(t_1)\rangle$. If we denote by $P(n_2, t_2|n_1, t_1)$ the non-stationary conditional probability density this reads explicitly:

$$\langle n(t_2)|n(t_1)\rangle = \sum_{n_2} n_2 P(n_2, t_2|n_1, t_1)$$

The correlation between $n(t_2)$ and $n(t_1)$ is given by:

$$\langle\langle(n(t_2)|n(t_1))(n(t_1)|n(t_0))\rangle\rangle = \sum_{n_2}\sum_{n_1} n_2 n_1 P(n_2, t_2|n_1, t_1) P(n_1, t_1|n_0, t_0)$$

$\langle\langle\cdot\rangle\rangle$ means averaging over all possible n_2 and over all possible n_1. We consider the two-stage protein expression model shown in Figure 1A of the main text. If the protein degradation rate d_1 is much lower than the mRNA degradation rate d_0 the conditional probability reads in zero-order approximation in $\gamma = d_0/d_1$[6]:

$$P(n_2, t_2|n_1, t_1) = \sum_{m=0}^{n_1} \frac{n_1! f(t_2 - t_1)^{n_1 - m}(1 - f(t_2 - t_1))^m}{(n_1 - m)! m!} P_0(n_2 - m, t_2 - t_1) + \mathcal{O}(\gamma^{-1}).$$

The function P_0 denotes the probability distribution for $n_1 = 0$ initial proteins given by:

$$P_0(n, t) = \frac{(a)_n}{n!}\left(\frac{b}{1+b}\right)^n \left(\frac{1 + ne^{-d_1 t}}{1+b}\right)^a \sum_{k=0}^{n} \frac{(-n)_k(-a)_k}{(1-a-n)_k k!}\left(\frac{(1+b)e^{-d_1 t}}{1+be^{-d_1 t}}\right)^k + \mathcal{O}(\gamma^{-1}),$$

where $(a)_n$ is the Pochhammer symbol[1], the parameters and the function f are defined as:

$$a = \frac{\nu_0}{d_1}, \quad b = \frac{\nu_1}{d_0}, \quad f(t) = 1 - e^{-d_1 t}. \tag{1}$$

In general the parameters will slightly vary from cell to cell and also over time (time-dependent extrinsic noise). For simplicity, we consider cell-to-cell variability in the transcription and translation rates ν_0 and ν_1 and keep the degradation rates constant (note

that in the simulations we only vary the translation rate ν_1). This means the transcription and translation rates vary from cell to cell, but are otherwise constant. To calculate the auto-correlation, one has to average over the extrinsic noise:

$$c(t_2, t_1; t_0) \equiv \frac{\langle\langle\langle(n(t_2)|n(t_1))(n(t_1)|n(t_0))\rangle\rangle\rangle_e - \langle\langle n(t_2)|n(t_0)\rangle\rangle_e \langle\langle n(t_1)|n(t_0)\rangle\rangle_e}{\sqrt{[\langle\langle(n(t_2)|n(t_0))^2\rangle\rangle_e - \langle\langle n(t_2)|n(t_0)\rangle\rangle_e^2]\,[\langle\langle(n(t_1)|n(t_0))^2\rangle\rangle_e - \langle\langle n(t_1)|n(t_0)\rangle\rangle_e^2]}}$$

Note that the above expression depends on the initial time t_0. Using the above stated approximations we find after some algebra for the auto-correlation:

$$c(t_2, t_1; t_0) = \frac{V^2 f_{20} f_{10} + \langle ab\rangle_e f_{10}(1 - f_{21}) + \langle ab^2\rangle_e g_{10}(1 - f_{21})}{\sqrt{\left[V^2 f_{20}^2 + \langle ab\rangle_e f_{20} + \langle ab^2\rangle_e g_{20}\right]\left[V^2 f_{10}^2 + \langle ab\rangle_e f_{10} + \langle ab^2\rangle_e g_{10}\right]}} + \mathcal{O}(\gamma^{-1}) \quad (2)$$

with

$$\begin{aligned} V^2 &= \langle a^2 b^2\rangle_e - \langle ab\rangle_e^2 \\ f_{pq} &= 1 - e^{-d_1(t_p - t_q)}, \quad g_{pq} = 1 - e^{-2d_1(t_p - t_q)} \end{aligned}$$

It is instructive to consider limiting cases:

- No extrinsic noise, $V^2 = 0$:

$$\lim_{V^2 \to 0} c(t_2, t_1; t_0) = \frac{f_{10}(1 - f_{21}) + (1 - f_{21}) b g_{10}}{\sqrt{[f_{20} + b g_{20}]\,[f_{10} + b g_{10}]}} + \mathcal{O}(\gamma^{-1})$$

- The limit $a \to \infty$, $b \to 0$ with ab finite corresponds to the one-stage gene expression model, simple production and degradation of a protein:

$$\lim_{\substack{ab=\text{const}\\ a\to\infty \\ b\to 0}} c(t_2, t_1; t_0) = \frac{V^2 f_{20} f_{10} + \langle ab\rangle_e f_{10}(1 - f_{21})}{\sqrt{\left[V^2 f_{20}^2 + \langle ab\rangle_e f_{20}\right]\left[V^2 f_{10}^2 + \langle ab\rangle_e f_{10}\right]}}$$

  This is the non-stationary autocorrelation for a death-birth process with extrinsic noise. It is exact, the correction of order $\mathcal{O}(\gamma^{-1})$ vanishes. To see why this is the case, write $a = \nu_0/d_0\gamma$ and $b = \nu_1/d_1\gamma^{-1}$ and take the limit $\gamma \to \infty$.

- Taking also the limit $V^2 \to 0$ yields:

$$\lim_{\substack{ab=\text{const}\\ a\to\infty \\ b\to 0}} \lim_{V^2 \to 0} c(t_2, t_1; t_0) = c_0(t_2, t_1; t_0) = \sqrt{\frac{f_{10}}{f_{20}}}(1 - f_{21})$$

  which is the non-stationary autocorrelation for a death-birth process with constant parameters. For $t_1 - t_0 \gg d_1^{-1}$ ($t_2 \geq t_1$) we obtain:

$$c_0(t_2, t_1) = (1 - f_{21}) = e^{-d_1(t_2 - t_1)}.$$

Note that c_0 only depends on the protein degradation rate.

The birth-death process as a lower bound

In the following we prove that the auto-correlation of the birth-death process provides a lower bound for the auto-correlation of the two-stage model with extrinsic noise. We start by rewriting Eq. (2):

$$
\begin{aligned}
c(t_2,t_1;t_0) &= c_0(t_2,t_1;t_0)\Gamma(t_2,t_1;t_0) \\
\Gamma(t_2,t_1;t_0) &= \frac{\frac{V^2}{(1-f_{21})\langle ab\rangle_e}f_{20}+1+\frac{\langle ab^2\rangle_e}{\langle ab\rangle_e}\frac{g_{10}}{f_{10}}}{\sqrt{\left[\frac{V^2}{\langle ab\rangle_e}f_{20}+1+\frac{\langle ab^2\rangle_e}{\langle ab\rangle_e}\frac{g_{20}}{f_{20}}\right]\left[\frac{V^2}{\langle ab\rangle_e}f_{10}+1+\frac{\langle ab^2\rangle_e}{\langle ab\rangle_e}\frac{g_{10}}{f_{10}}\right]}}
\end{aligned}
$$

It is sufficient to show that $\Gamma \geq 1$ for all possible parameter combinations. Because $1-f_{21} \leq 1$ we have:

$$
\Gamma(t_2,t_1;t_0) \geq \frac{\frac{V^2}{\langle ab\rangle_e}f_{20}+1+\frac{\langle ab^2\rangle_e}{\langle ab\rangle_e}\frac{g_{10}}{f_{10}}}{\sqrt{\left[\frac{V^2}{\langle ab\rangle_e}f_{20}+1+\frac{\langle ab^2\rangle_e}{\langle ab\rangle_e}\frac{g_{20}}{f_{20}}\right]\left[\frac{V^2}{\langle ab\rangle_e}f_{10}+1+\frac{\langle ab^2\rangle_e}{\langle ab\rangle_e}\frac{g_{10}}{f_{10}}\right]}}
$$

Further, because $f_{20} \geq f_{10}$ $(t_2 \geq t_1)$ we find:

$$
\Gamma(t_2,t_1;t_0) \geq \frac{\frac{V^2}{\langle ab\rangle_e}f_{20}+1+\frac{\langle ab^2\rangle_e}{\langle ab\rangle_e}\frac{g_{10}}{f_{10}}}{\sqrt{\left[\frac{V^2}{\langle ab\rangle_e}f_{20}+1+\frac{\langle ab^2\rangle_e}{\langle ab\rangle_e}\frac{g_{20}}{f_{20}}\right]\left[\frac{V^2}{\langle ab\rangle_e}f_{20}+1+\frac{\langle ab^2\rangle_e}{\langle ab\rangle_e}\frac{g_{10}}{f_{10}}\right]}}
$$

We have

$$
\begin{aligned}
\frac{g_{20}}{f_{20}} &\leq \frac{g_{10}}{f_{10}} \Leftrightarrow g_{20}f_{10} \leq g_{10}f_{20} \Leftrightarrow (1-(1-f_{20})^2)f_{10} \leq (1-(1-f_{10})^2)f_{20} \\
\Leftrightarrow f_{10}f_{20}^2 &\geq f_{10}^2 f_{20} \Leftrightarrow f_{20} \geq f_{10}
\end{aligned}
$$

and with this

$$
\Rightarrow \Gamma(t_2,t_1;t_0) \geq \frac{\frac{V^2}{\langle ab\rangle_e}f_{20}+1+\frac{\langle ab^2\rangle_e}{\langle ab\rangle_e}\frac{g_{10}}{f_{10}}}{\sqrt{\left[\frac{V^2}{\langle ab\rangle_e}f_{20}+1+\frac{\langle ab^2\rangle_e}{\langle ab\rangle_e}\frac{g_{10}}{f_{10}}\right]\left[\frac{V^2}{\langle ab\rangle_e}f_{20}+1+\frac{\langle ab^2\rangle_e}{\langle ab\rangle_e}\frac{g_{10}}{f_{10}}\right]}} = 1.
$$

It follows that $1 \geq c(t_2,t_1;t_0) \geq c_0(t_2,t_1;t_0)$.

Simulation of the KikGR experiment

In order to test our analytical results we simulated the KikGR experiment. For sake of simplicity we kept all parameters constant besides the translational rate ν_1. We simulated 10^5 trajectories of a single reporter according to:

1. Draw the translation rate ν_1 from a log-normal distribution with $\langle \nu_1 \rangle = 45\,\mathrm{h}^{-1}$ and $\mathrm{Var}(\nu_1) \in \{0\,\mathrm{h}^{-2}, 10^2\,\mathrm{h}^{-2}\}$.
2. Simulate the reporter until stationary state with zero protein initial condition and mRNA initial condition drawn from the Poisson distribution
$$P(m) = \frac{\left(\frac{\nu_0}{d_0}\right)^m}{m!} e^{-\frac{\nu_0}{d_0}}.$$
3. Compute the non-stationary auto-correlation.

A typical simulation result is shown in Figure 1B. After the bleach at time $t_0 = 0$ the red proteins decay and the green proteins build up until being in stationary state. In Figure 1C we compare c_0 to the simulation results for different CVs of the protein translation rate ν_1. In Supplementary Figure 24A we compare the zero-order approximation for the auto-correlation given in Eq. (2) with simulation results for finite γ. We also show c_0 as the lower bound of the auto-correlation. The dependency of the non-stationary auto-correlation on t_1 can be seen in Supplementary Figure 24B ($\gamma = 12.5$) and Supplementary Figure 24C ($\gamma = 1250$), respectively.

Estimation of the protein half-life

In order to obtain an estimate for c_0 we estimated the protein degradation rate by measuring the fluorescent of the remaining red fluorescence proteins after conversion. Note that we were only interested in obtaining a rough estimate of the lifetime of the protein. For simplicity we ignored bleaching effects; by this we may overestimate the degradation rate, by means of which our estimate is an upper bound of the true rate. To obtain the estimate we processed the data as follows. For each cell we have three measurements: x_0 the fluorescence directly after the conversion ($t_0 = 0$), x_1 ($t_1 = 3\,\text{h}$) and x_2 ($t_2 = 6\,\text{h}$) after the conversion. The amount of red proteins at time $t \geq 0$ is given by $x(t) = x_0 e^{-d_1 t}$. We transformed the data logarithmically and calculated for each cell an optimal degradation value by minimising:

$$L = \left(\ln \left[\frac{x_1}{x_0} \right] + d_1 t_1 \right)^2 + \left(\ln \left[\frac{x_2}{x_0} \right] + d_1 t_2 \right)^2 \tag{3}$$

with respect to d_1. Because the logarithm is a strictly monotonic function this transformation preserves the optimum in absence of noise and measurements errors, but simplifies its calculation considerably. As long as the noise is small the optima (with and without transformation) are very similar. We incur for the optimal d_1:

$$d_1 = -\frac{t_1 \ln \left[\frac{x_1}{x_0} \right] + t_2 \ln \left[\frac{x_2}{x_0} \right]}{t_1^2 + t_2^2}.$$

We find for d_1: $(d_1)_{0.5} = 0.095\,\text{h}^{-1}$ (median), $\bar{d_1} = 0.091\,\text{h}^{-1}$ (mean), and $\sigma = 0.023\,\text{h}^{-1}$ (SD). Using $d_1 = 0.09\,\text{h}^{-1}$ together with $t_2 = 6$ h, $t_1 = 3$ h and $t_0 = 0$ h in the expression for the auto-correlation we find $c_0 \approx 0.6$. Thus the theoretical prediction for the auto-correlation is $1 \geq c \geq 0.6$.

Supplementary Note 2: Spatial correlation

Diffusion-like transport

Movement of molecules between cells can induce a spatial correlation. To see this, we briefly investigate a simplified system. We consider a protein which synthesized with rate σ_i in cell i, is degraded with rate λ and is transported across cell boundaries with rate μ. We assume that μ and λ are homogeneous and constant over the grid. The synthesis, however, are stochastic variables. For sake of simplicity, we consider them to be time independent, i.e., the synthesis rates σ_i vary from cell to cell, but are otherwise constant. The concentration of the protein on the tissue can be described by:

$$\frac{dx}{dt} = (\mu C - \lambda \mathbb{1})x + b$$

where x is a vector of the concentrations of the protein on the cellular grid, C is the connectivity matrix of the grid and b is a vector with the production rates σ_i. The connectivity matrix is given by $C_{ii} = -|\Omega_i|$ and $C_{ij} = 1$ for $i \neq j$ if cell i is connected to cell j and $C_{ij} = 0$ otherwise. $|\Omega_i|$ denotes the number of connected neighbors of cell i. For low mobility the contribution to the correlation due protein mobility will be proportional to $\varepsilon = \mu/\lambda$. This can be seen as follows. In steady state the solution for x can be written formally:

$$x = (\mathbb{1} - \varepsilon C)^{-1}\tilde{b}$$

with $\tilde{b} = \lambda^{-1}b$. In case $\varepsilon \ll 1$ we can expand x in terms of ε:

$$x = \sum_{n=0}^{N} \varepsilon^n C^n \tilde{b},$$

(N is the number of cells on the grid) from which follows:

$$x_i = \tilde{b}_i + \varepsilon \sum_j^N C_{ij}\tilde{b}_j + \mathcal{O}(\varepsilon^2).$$

For the covariance between two cells we find:

$$\mathrm{cov}(x_i, x_j) = \mathrm{cov}(\tilde{b}_i, \tilde{b}_j) + \varepsilon \left[2V^2 + \sum_{n \neq j}^{N} C_{in}\mathrm{cov}(\tilde{b}_j, \tilde{b}_n) + \sum_{m \neq i}^{N} C_{jm}\mathrm{cov}(\tilde{b}_i, \tilde{b}_m) \right] + \mathcal{O}(\varepsilon^2).$$

In case of no spatial correlation between the synthesis rates all $\mathrm{cov}(\tilde{b}_n, \tilde{b}_m)$ vanish; however, the covariance between two cells would be non-zero and proportional to ε ($V^2 = \langle b_i^2 \rangle - \langle b_i \rangle^2$, $\forall i \in \{1, ..., N\}$). Since we are interested in the covariance between the expression

systems of two neighbouring cells (i.e., $\mathrm{cov}(\tilde{b}_n, \tilde{b}_m)$), it is important to find an estimate for ε. To obtain this we consider the case in which we have only one cell producing the protein (the donor cell), all other cells are receptor cells, i.e., $\tilde{b}_1 = \sigma/\lambda$, $\tilde{b}_i = 0$ for $i \neq 1$. In this case we have: $x_1 = \tilde{b}_1 - \varepsilon|\Omega_1|\tilde{b}_1$ and $x_i = \varepsilon\tilde{b}_1$, from which follows:

$$\frac{x_i}{x_1} = \varepsilon + \mathcal{O}(\varepsilon^2).$$

By building the ratio of the measured fluorescence of a receptor cell to the measured fluorescence of the donor cell, i.e., by determining the fraction of fluorescence obtained by the receptor cell, one can find an approximation for ε.

Characterization of localization and movement of YFP tags

We aimed to choose CFP and YFP tags that minimize intercellular movement and that localize completely to the nucleus to enable accurate measurements. We compared three tags: YFP, 2xNLS-YFP and NLS-2xYFP that was published to be cell-autonomous. Arabidopsis leaves were transiently transformed by biolistic transformations. Free YFP was localized in the cytoplasm and the nucleus (Supplementary Figure 1A). As compared to NLS-2xYFP, 2xNLS-YFP showed a stronger nuclear localization, while NLS-2xYFP was still prominently localized in the cytoplasm (Supplementary Figure 1B, C). Unexpectedly, all YFP variants tested showed the ability to move to adjacent cells. To obtain an estimate for ε we have to build the ratio between the donor and the receptor cells. A precise value for ε would be difficult to obtain, but for our purpose it is sufficient to obtain an upper bound. This simplifies the experimental analysis considerably. For each donor cell we only determine the ratio between the receptor cell with the highest fluorescence and the donor cell. This systematically overestimate the rate, but provides a sufficient upper bound. Free YFP showed the highest movement ($\varepsilon = 0.08$ $(+0.019/-0.022)$, basic bootstrap confidence intervalls). 2xNLS-YFP ($\varepsilon = 0.0218$ $(+0.0061/-0.0066)$) and NLS-2xYFP ($\varepsilon = 0.0047$ $(+0.0016/-0.0018)$) both revealed much lower values (Supplementary Figure 1D). We decided to use 2xNLS-YFP for our analysis of the spatial correlation as this fusion exhibits only slightly higher movement rates, but enabled us to precisely measure the fluorescence in individual cells due to its strong nuclear localization.

Pearson correlation between neighbouring cells

The half-life of the used reporter CFP and YFP (24 h)[7] is of the same order as the cell division time of epidermal cells on young leaves (33 h)[5]. Due to this the stochastic gene expression system is never in stationary state on young growing leaves. This gives rise to an extra term when calculating the covariance from a single reporter. The daughter cells inherit mRNA and protein from their mother cell, by which the initial conditions are the same (or at least very similar). In order to analyse the consequence of this we write:

$$\mathrm{cov}(x_1, x_2) \quad = \quad \langle\langle\langle(x_1|N_1, z_1)(x_2|N_2, z_2)\rangle\rangle_N\rangle_e - \langle\langle\langle x_1|N_1, z_1\rangle\rangle_N\rangle_e\langle\langle\langle x_2|N_2, z_2\rangle\rangle_N\rangle_e$$

where N_1 and N_2 denote the protein amounts directly after cell division and z_1 and z_2 denote the extrinsic stochastic processes in cell 1 and cell 2, resp. $\langle\cdot\rangle_N$ means averaging over all possible initial conditions. We assume that the intrinsic processes in cell 1 and cell 2 are independent in a mechanistic sense, i.e., they do not influence each other in any way (directly or indirectly through feedbacks to the environment), which implies they are statistically independent for a given realisation of sample extrinsic noise z_1 and z_2. Further, we assume the stochastic processes are the same in all cells, i.e., $\langle\langle\langle x_1|N_1, z_1\rangle\rangle_N\rangle_e \equiv \langle\langle\langle x_2|N_2, z_2\rangle\rangle_N\rangle_e \equiv \langle\langle\langle x|N, z\rangle\rangle_N\rangle_e$. Therefore, we can rewrite the covariance:

$$\mathrm{cov}(x_1, x_2) \quad = \quad \langle\langle\langle x_1|N_1, z_1\rangle\langle x_2|N_2, z_2\rangle\rangle_N\rangle_e - \langle\langle\langle x|N, z\rangle\rangle_N\rangle_e^2$$

We investigate now the case where the stochastic extrinsic processes z_i and z_j are identical. If the initial conditions N_1 and N_2 are independent we find:

$$\mathrm{cov}(x_1, x_2) = \langle\langle\langle x|N, z\rangle\rangle_N^2\rangle_e - \langle\langle\langle x|N, z\rangle\rangle_N\rangle_e^2 = \sigma_{\mathrm{ext}}^2. \tag{4}$$

However, if the initial conditions are identical as it is in case of cell division, one obtains (again for identical z_1 and z_2):

$$\mathrm{cov}(x_1, x_2) = \langle\langle\langle x|N, z\rangle^2\rangle_N\rangle_e - \langle\langle\langle x|N, z\rangle\rangle_N\rangle_e^2 \neq \sigma_{\mathrm{ext}}^2. \tag{5}$$

In a growing tissue there will be always next neighbour cells which are daughter cells and others which are not. Note that in stationary state the expressions given in Eq. (4) and (5) are identical, because all dependence on the initial conditions is decayed. Since on young developing leaves the gene expression systems are not in stationary state it is better to estimate the spatial correlation of the extrinsic noise by using the dual reporter system correlating CFP from cell 1 to YFP in cell 2:

$$\mathrm{cov}(c_1, y_2) \quad = \quad \langle\langle\langle c_1|N_1, z_1\rangle\langle y_2|N_2, z_2\rangle\rangle_N\rangle_e - \langle\langle\langle c|N, z\rangle\rangle_N\rangle_e\langle\langle\langle y|N, z\rangle\rangle_N\rangle_e$$

with c_i and y_i denoting the two channels of the dual reporter system of cell i. The initial mRNA and protein abundance of CFP are identical in the two daughter cells and the same

is true for YFP, but the initial conditions of CFP are different and independent of YFP and vice versa. We arrive at:

$$\mathrm{cov}(c_1, y_2) \;=\; \langle\langle\langle c_1|N_1, z_1\rangle\rangle_N \langle\langle y_2|N_2, z_2\rangle\rangle_N\rangle_e - \langle\langle\langle c|N, z\rangle\rangle_N\rangle_e \langle\langle\langle y|N, z\rangle\rangle_N\rangle_e \qquad (6)$$

which yields for identical stochastic extrinsic processes z_1 and z_2 Eq. (4). We therefore define Pearson's correlation coefficient between two neighbouring cells:

$$r = \frac{\mathrm{cov}(c_1, y_2)}{\sigma^2_{\mathrm{ext}}}. \qquad (7)$$

To analyse the experimental data we define for each set of cells from a given leaf: $\hat{c} = \frac{1}{N}\sum_{i=1}^{N} x_i$, $\hat{y} = \frac{1}{N}\sum_{i=1}^{N} y_i$, $\hat{\sigma}^2_{\mathrm{ext}} = \frac{1}{N-1}\sum_{i=1}^{N}(c_i - \hat{x})(y_i - \hat{y})$, and

$$r \;=\; \frac{1}{2\hat{\sigma}^2_{\mathrm{ext}} \sum_{i=1}^{N} |\Omega_i|} \sum_{i=1}^{N} \sum_{j\in\Omega_i} (c_i - \hat{c})(y_j - \hat{y}) + (y_i - \hat{y})(c_j - \hat{c}) \qquad (8)$$

where N are the number of cells on the particular leave, the neighbours of cell i are given by the set Ω_i and the number of neighbours by $|\Omega_i|$. Note that r is also scale invariant, i.e., a scaling factor between c and y does not change r.

Supplementary Note 3: Measuring intrinsic and extrinsic noise

In presence of intrinsic and extrinsic noise the total variance of a stochastic component x can be decomposed[4;2]: $\sigma^2 = \sigma^2_{\text{int}} + \sigma^2_{\text{ext}}$ with:

$$\begin{aligned}\sigma^2_{\text{int}} &= \langle\langle x^2|z\rangle\rangle_e - \langle\langle x|z\rangle^2\rangle_e \\ \sigma^2_{\text{ext}} &= \langle\langle x|z\rangle^2\rangle_e - \langle\langle x|z\rangle\rangle^2_e\end{aligned}$$

z denotes the extrinsic stochastic process and the outer brackets indicate averages over all states of z (extrinsic noise). Using the dual reporter system (with c and y denoting the reporter abundance) we can write:

$$\langle\langle (c-y)^2|z\rangle\rangle_e = \langle\langle c^2|z\rangle\rangle_e + \langle\langle y^2|z\rangle\rangle_e - 2\langle\langle c|z\rangle\langle y|z\rangle\rangle_e$$
$$\langle\langle cy|z\rangle\rangle_e - \langle\langle c|z\rangle\rangle_e\langle\langle y|z\rangle\rangle_e = \langle\langle c|z\rangle\langle y|z\rangle\rangle_e - \langle\langle c|z\rangle\rangle_e\langle\langle y|z\rangle\rangle_e$$

If the reporters are identical and independent we have:

$$\langle\langle (c-y)^2|z\rangle\rangle_e = 2\langle\langle c^2|z\rangle\rangle_e - 2\langle\langle c|z\rangle^2\rangle_e = 2\sigma^2_{\text{int}}$$
$$\langle\langle cy|z\rangle\rangle_e - \langle\langle c|z\rangle\rangle_e\langle\langle y|z\rangle\rangle_e = \langle\langle c|z\rangle^2\rangle_e - \langle\langle c|z\rangle\rangle^2_e = \sigma^2_{\text{ext}}.$$

Using the definition for the intrinsic and extrinsic noise:

$$\eta^2_{\text{int}} = \frac{\sigma^2_{\text{int}}}{\langle c\rangle^2}, \quad \eta^2_{\text{ext}} = \frac{\sigma^2_{\text{ext}}}{\langle c\rangle^2}$$

we arrive at the expressions[3]:

$$\eta^2_{\text{int}} = \frac{\langle (c-y)^2\rangle}{2\langle c\rangle\langle y\rangle}, \quad \eta^2_{\text{ext}} = \frac{\langle cy\rangle - \langle c\rangle\langle y\rangle}{\langle c\rangle\langle y\rangle}. \tag{9}$$

We used $\langle\cdot\rangle$ as a short hand notation for the average over extrinsic and intrinsic stochasticity.

In the experiment one cannot measure the abundance of the reporter directly, but rather quantifies the fluorescence. When marking the region of interest (ROI) one may mark pixels which do not belong to the true ROI or miss pixels which do. If we denote the average intensity of the true ROI with N pixels as c and the average intensity of the extra or missed n pixels as s (note that n can be positive or negative) we can write for the measured signal m:

$$m = p\frac{Nc + ns}{N+n} \tag{10}$$

where p is the proportionally factors between fluorescence and abundance. The average intensity s of the extra or missed pixels is related to the average intensity c of the nucleus.

We therefore write $s = c(1+\eta)$ where η is a stochastic number with $|\eta| < 1$, $\langle c\eta\rangle = \langle c\rangle\langle\eta\rangle$ and $\langle\eta\rangle = 0$ ($\Rightarrow \langle s\rangle = \langle c\rangle$). With this we rewrite Eq. (10):

$$m = pc\,(1+\varepsilon_m)\,, \tag{11}$$

with the definition:

$$\varepsilon_m = \frac{\frac{n}{N}}{1+\frac{n}{N}}\eta. \tag{12}$$

It is important to realise that n, the number of erroneously marked or missed pixels and s the average intensity of these pixels are uncorrelated stochastic numbers. Due to this we obtain $\langle\varepsilon_m\rangle = 0$ and $\langle m\rangle = p\langle c\rangle$. Altogether, we can write for the measured CFP and YFP signals:

$$m_c = p_c c(1+\varepsilon_m) + \varepsilon_c \tag{13}$$

$$m_y = p_y y(1+\varepsilon_m) + \varepsilon_y. \tag{14}$$

ε_c and ε_y denote the technical error due to stochasticity of the equipment (in general different for c and y). Because there is no bias in the experiment $\langle\varepsilon_c\rangle = \langle\varepsilon_y\rangle = 0$ holds. The proportionally factors between fluorescence and abundance p_c and p_y are in general different. This causes a problem, when one estimates the intrinsic noise from data. To see this we ignore the errors in Eqs. (13) and (14) and calculate the intrinsic noise using Eq. (9):

$$\frac{\langle(m_c - m_y)^2\rangle}{2\langle m_c\rangle\langle m_y\rangle} = \frac{p_c}{p_y}\frac{\left\langle\left(c - \frac{p_y}{p_c}y\right)^2\right\rangle}{2\langle c\rangle\langle y\rangle} \neq \frac{\langle(c-y)^2\rangle}{2\langle c\rangle\langle y\rangle}. \tag{15}$$

We therefore normalise the data with the mean and again (for the sake of the argument) ignore the errors:

$$\frac{\langle(m_c/\langle m_c\rangle - m_y/\langle m_y\rangle)^2\rangle}{2} = \frac{\langle(c/\langle c\rangle - y/\langle y\rangle)^2\rangle}{2} \overset{\langle c\rangle=\langle y\rangle}{=} \frac{\langle(c-y)^2\rangle}{2\langle c\rangle\langle y\rangle}$$

The last equality holds if $\langle c\rangle = \langle y\rangle$, which is true for the dual reporter system. In presence of technical and measurement errors the estimated (apparent) intrinsic noise is given by:

$$\eta^2_{\text{int,app}} = \frac{\langle(m_c/\langle m_c\rangle - m_y/\langle m_y\rangle)^2\rangle}{2} = \eta^2_{\text{int}}\left(1 + \langle\varepsilon_m^2\rangle\right) + \mathcal{E}_t^2$$

with the total technical error:

$$\mathcal{E}_t^2 = \frac{\langle\varepsilon_c^2\rangle}{2\langle m_c\rangle^2} + \frac{\langle\varepsilon_y^2\rangle}{2\langle m_y\rangle^2}.$$

For the estimated (apparent) extrinsic noise we find:

$$\eta^2_{\text{ext,app}} = \frac{\langle m_c m_y\rangle - \langle m_c\rangle\langle m_y\rangle}{\langle m_c\rangle\langle m_y\rangle} = \eta^2_{\text{ext}}\left(1 + \langle \varepsilon^2_m\rangle\right). \tag{16}$$

In both cases the apparent noise overestimates the true intrinsic and extrinsic noise, respectively. The factors (relative error) by which the intrinsic and extrinsic noise is overestimated are given by:

$$f_{\text{int}} = \frac{\eta_{\text{int,app}}}{\eta_{\text{int}}} - 1 = \sqrt{\frac{1+\langle\varepsilon^2_m\rangle}{1-\frac{\mathcal{E}^2_t}{\eta^2_{\text{int,app}}}}} - 1, \quad f_{\text{ext}} = \frac{\eta_{\text{ext,app}}}{\eta_{\text{ext}}} - 1 = \sqrt{1+\langle\varepsilon^2_m\rangle} - 1 \tag{17}$$

In order to estimate the technical error generated by the used instruments ($\langle\varepsilon^2_c\rangle$, $\langle\varepsilon^2_y\rangle$), a yellow autofluorescent plastic slide (ChromaTechnologies, https://www.chroma.com/products/accessories/92001-autofluorescent-plastic-slides) was imaged with identical CFP and YFP settings and laser intensities as used for noise measurements. Because fluorochromes do not bleach and diffuse in this type of slides, only one region of interest was imaged 221 times to avoid variation in our measurements due to inhomogeneities. Because we do not have any additional errors besides the technical error we can write: $m^s_c = p^s_c s + \varepsilon_c$ and $m^s_y = p^s_y s + \varepsilon_y$, where s is the signal from the yellow autofluorescent plastic slide. We find:

$$\frac{\text{Var}(m^s_c)}{\langle m^s_c\rangle^2} = \frac{\text{Var}(s)}{\langle s\rangle^2} + \frac{\langle\varepsilon^2_c\rangle}{\langle m^s_c\rangle^2}, \quad \frac{\text{Var}(m^s_y)}{\langle m^s_y\rangle^2} = \frac{\text{Var}(s)}{\langle s\rangle^2} + \frac{\langle\varepsilon^2_y\rangle}{\langle m^s_y\rangle^2}$$

and

$$\langle(m^s_c/\langle m^s_c\rangle - m^s_y/\langle m^s_y\rangle)^2\rangle = \frac{\langle\varepsilon^2_c\rangle}{\langle m^s_c\rangle^2} + \frac{\langle\varepsilon^2_y\rangle}{\langle m^s_y\rangle^2}.$$

Taken together we obtain estimates for the technical errors:

$$\frac{\text{Var}(s)}{\langle s\rangle^2} = 3.7\times10^{-6}, \quad \frac{\langle\varepsilon^2_c\rangle}{\langle m^s_c\rangle^2} = 1.6\times10^{-5}, \quad \frac{\langle\varepsilon^2_y\rangle}{\langle m^s_y\rangle^2} = 9.6\times10^{-7}. \tag{18}$$

In contrast to the technical errors coming from the microscopy equipment the measurement error is the same for both channels per analysed cell. To obtain an estimate for ε_m, we performed the following series of experiments. The ROI were on three leaves marked three times. This means that the technical errors and the CFP and YFP amount are constant for the three subsequent measurements of the nuclei. The estimator for covariance between the measured signal for CFP and YFP reads:

$$\widehat{\text{cov}}(m^n_c, m^n_y) = \frac{1}{2}\sum_{i=1}^{3}\left(m^{in}_c - \frac{1}{3}\sum_{i=1}^{3} m^{in}_c\right)\left(m^{in}_y - \frac{1}{3}\sum_{i=1}^{3} m^{in}_y\right). \tag{19}$$

$m^{in}_{c/y}$ are the signals obtained from the ith measurements of the nth nucleus. Using Eqs. (13) and (14) we find:

$$\widehat{\text{cov}}(m^n_c, m^n_y) = \frac{p_c p_y c_n y_n}{2} \sum_{i=1}^{3} \left(\varepsilon_{m,in} - \frac{1}{3} \sum_{i=1}^{3} \varepsilon_{m,in} \right) \left(\varepsilon_{m,in} - \frac{1}{3} \sum_{i=1}^{3} \varepsilon_{m,in} \right). \tag{20}$$

It follows for the average over sufficiently many nuclei:

$$\frac{1}{N} \sum_{n}^{N} \widehat{\text{cov}}(m^n_c, m^n_y) = \left\langle \widehat{\text{cov}}(m^n_c, m^n_y) \right\rangle = p_c p_y \langle cy \rangle \langle \varepsilon^2_m \rangle. \tag{21}$$

We also find:

$$\langle m_c m_y \rangle = \frac{1}{3N} \sum_{n=1}^{N} \sum_{i=1}^{3} m^{in}_c m^{in}_y = p_c p_y \, \langle (c(1+\varepsilon_m))(y(1+\varepsilon_m)) \rangle = p_c p_y \langle cy \rangle (1 + \langle \varepsilon^2_m \rangle). \tag{22}$$

From Eqs. (21) and (22) one obtains:

$$\langle \varepsilon^2_m \rangle = \frac{\left\langle \widehat{\text{cov}}(m^n_c, m^n_y) \right\rangle}{\langle m_c m_y \rangle - \left\langle \widehat{\text{cov}}(m^n_c, m^n_y) \right\rangle} \tag{23}$$

We estimate $\langle \varepsilon^2_m \rangle$ by averaging over 100 nuclei on 3 different leaves (*i.e.*, 300 nuclei in total):

$$\langle \varepsilon^2_m \rangle = 4.0 \times 10^{-3}. \tag{24}$$

The measurement noise is two orders of magnitude larger than the technical errors from the microscopy equipment and thus dominates the relative errors made by estimating the intrinsic and extrinsic noise. The error of the extrinsic noise is the same for all measurements while the error of the intrinsic noise depends on the actual measured apparent intrinsic noise (Eq. 17). We find $f_{\text{ext}} = 2.0 \times 10^{-3}$ and $f_{\text{int}} < 2.1 \times 10^{-3}$ for all measured values of the intrinsic noise. We conclude that in both cases the error introduced due to mistakes made by marking of the ROI and fluctuations in the technical equipment are negligible.

Supplementary Note 4: Spatial correlation due to cell division

When cells divide the mRNA and protein content of the mother cell is inherited to the daughter cells. When calculating the CFP-YFP cross-correlation of the dual reporter system one avoids to introduce a correlation due to identical initial conditions. However, even though the initial conditions of CFP and YFP of the daughter cells are different they come from the same distribution, the underlying protein distribution of the mother cell. This induces a correlation which will decay with time. To show this we start with Eq. (6) and make the dependence on the extrinsic noise process z_0 of the mother cell explicit:

$$\begin{aligned}\mathrm{cov}(c_1, y_2) &= \langle\langle\langle\langle\langle c_1|N_1, z_1, z_0\rangle\rangle_N \langle\langle y_2|N_2, z_2, z_0\rangle\rangle_N\rangle_{z_1}\rangle_{z_2}\rangle_{z_0} \\ &\quad -\langle\langle\langle\langle c|N, z, z_0\rangle\rangle_N\rangle_z\rangle_{z_0}\langle\langle\langle\langle y|N, z, z_0\rangle\rangle_N\rangle_z\rangle_{z_0}\end{aligned}$$

If we assume for simplicity that z_0, z_1 and z_2 are independent we find:

$$\mathrm{cov}(c_1, y_2) = \langle\langle\langle\langle x|N, z\rangle\rangle_N\rangle_z^2\rangle_{z_0} - \langle\langle\langle x|N, z\rangle\rangle_N\rangle_z\rangle_{z_0}^2.$$

At $t = 0$, right after cell division the expression above yields σ^2_{ext}, while for $t \to \infty$ the gene expression of the daughter cells are independent from the stochastic processes in the mother cell, therefore expect we $\mathrm{cov}(c_1, y_2) \to 0$ for large t. It follows that for the correlation (defined by Eq. 7) between daughter cells $r(t) \leq 1$ with $r(t = 0) = 1$ and $\lim_{t\to\infty} r(t) = 0$ holds. To determine $r(t)$ we calculate the non-stationary covariance between two daughter cells as well as σ^2_{ext} using the two-stage gene expression model. Because we only wish to estimate the contribution of cell division to the overall spatial correlation, we employ a simple cell division model. At time $t = 0$ an exact copy of the mother cell is produced. Thereby we avoid all complication introduced by cell growth. We obtain:

$$\sigma^2_{\mathrm{ext}}(t) = \left(\langle a^2b^2\rangle - \langle ab\rangle^2\right)\left(1 - 2e^{-d_1t} + 2e^{-2d_1t}\right) \quad (25)$$

$$\mathrm{cov}(c_1, y_2)(t) = \left(\langle a^2b^2\rangle - \langle ab\rangle^2\right)e^{-2d_1t} \quad (26)$$

$$r(t) = \frac{e^{-2d_1t}}{(1 - 2e^{-d_1t} + 2e^{-2d_1t})}. \quad (27)$$

Simulation of the stochastic gene expression in daughter cells

We calculate the correlation between two cells that originate from the same mother cell. The daughter cells inherit all rates from the mother cell, beside the translational rate ν_1. In order to estimate how much of the correlation between cells stems from mRNA and protein inheritance, we assume that the translation rates of the daughter cells are in general different to each other and also differ from the translational rate of the mother cell. The dual reporter system is simulated as follows:

1. Draw the translation rate ν_1^0 of the mother cell from a log-normal distribution with $\langle \nu_1^0 \rangle = 500\,\mathrm{h}^{-1}$ and $\mathrm{Var}(\nu_1^0) = 1000\,\mathrm{h}^{-2}$.

2. mRNA and protein of CFP and YFP of the mother cell are drawn from the steady state distributions of the two-stage gene expression model[6]. We set $\gamma = 12.5$ and for this the deviations of the analytical protein steady state distribution and the true are small[6].

3. Draw the translation rates ν_1^1 and ν_1^2 of the daughters cell from a log-normal distribution with $\langle \nu_1^1 \rangle = \langle \nu_1^2 \rangle = \nu_1^0$ and $\mathrm{Var}(\nu_1^1) = \mathrm{Var}(\nu_1^2) = 1000\,\mathrm{h}^{-2}$.

4. Simulate the dual reporter system until stationary state.

This process is repeated 10^6 times. At each interval of $\Delta t = 0.01$, we compute the Pearson correlation according to Eq. (8). Typical trajectories of CFP and YFP for mother and daughter cells are shown in Supplementary Figure 25A. After cell division the trajectories for CFP and YFP become different because of the different translational rates (extrinsic noise) and the stochasticity of the gene expression systems itself (intrinsic noise). From the simulation we estimate the temporal correlation between the two daughter cells. The results are shown in Supplementary Figure 25B, which are in agreement with the theoretical predictions.

Estimating the contribution of cell division to the measured next neighbour correlation

As shown above cell division introduces a correlation between neighbouring cells. This means that even though there are no further correlated processes, one may find a correlation due to inheritance of mRNA and protein. It is therefore important to estimate the value of this contribution. When we calculate the correlation between cells and their next neighbours we do not know which cells are daughter cells, but to make progress we reason that for any given cell two cells out of its neighbouring cells are progeny cells from the last two cell division events. Because we also do not know when after the cell division we observe the cells, we argue that all times within the intervals $[0, T]$ for the last cell division and $[T, 2T]$ from the next to last cell division are equally likely, where T is the inverse cell division rate. For young leaves we have $T = 33$ h[5]. We average $r(t)$ over these time intervals:

$$r_1 = \frac{1}{T}\int_0^T r(t)dt, \quad r_2 = \frac{1}{T}\int_T^{2T} r(t)dt$$

and find $r_1 = 0.69$ and $r_2 = 0.11$. We used $d_1 = 0.029\ \mathrm{h}^{-1}$ as the degradation rate for CFP and YFP[7]. On average the cells have five neighbouring cells. Given that two

from these five cells are correlated to the center cell through inheritance of mRNA and protein content and the others are uncorrelated we arrive at our final estimate for the contribution of cell division to the measure next-neighbour correlation: $r = (r_1 + r_2)/5 \approx 0.16$.

If the daughter cells inherit not only the mRNA and protein content but also all other relevant features from the mother cell, the three cells have the same rates. In this case the correlation between the cells is always one, given the rates are constant over time. Following the same argument as outlined above one would expect a total next neighbour correlation of $r = 0.4$.

Supplementary Table 1: List of constructs used in this study.

Main figures

	Construct	Tissue	Transformation
Figure 1	*p35S:NLS-KikGR*<br>*pUBQ10:NLS-KikGR*	young leaf	stable
Figure 2	Simulation	-	-
Figure 3	*p35S:2xNLS-YFP p35S:2xNLS-CFP* line#1	young leaf<br>mature leaf	stable
Figure 4	*p35S:2xNLS-YFP p35S:2xNLS-CFP* line#1	primary root tip<br>hypocotyl<br>stomata (mature leaf)	stable
Figure 5	*p35S:2xNLS-YFP p35S:2xNLS-CFP* line#1<br>*pUBQ10:2xNLS-YFP pUBQ10:2xNLS-CFP*	young leaf<br>mature leaf<br>young leaf<br>mature leaf	stable

Supplementary figures

	Construct	Tissue	Transformation
Figure S1	*p35S:YFP*<br>*p35S:2xNLS-YFP*<br>*p35S:NLS-2xYFP*<br>*p35S:NLS-KikGR*	mature leaf	transient
Figure S2	*p35S:NLS-KikGR*	young leaf	stable
Figure S3	*pUBQ10:NLS-KikGR*	young leaf	stable
Figure S4	*p35S:NLS-KikGR*	young leaf	stable
Figure S5	*p35S:2xNLS-YFP p35S:2xNLS-CFP* line#1	young leaf	stable
Figure S6	*p35S:2xNLS-YFP p35S:2xNLS-CFP* line#1	mature leaf	stable
Figure S7	*p35S:2xNLS-YFP p35S:2xNLS-CFP* line#2<br>*pUBQ10:2xNLS-YFP pUBQ10:2xNLS-CFP*	young leaf<br>mature leaf<br>young leaf<br>mature leaf	stable
Figure S8	*p35S:2xNLS-YFP p35S:2xNLS-CFP* line#2	young leaf	stable
Figure S9	*p35S:2xNLS-YFP p35S:2xNLS-CFP* line#2	mature leaf	stable
Figure S10	*pUBQ10:2xNLS-YFP pUBQ10:2xNLS-CFP*	young leaf	stable
Figure S11	*pUBQ10:2xNLS-YFP pUBQ10:2xNLS-CFP*	mature leaf	stable
Figure S12	*p35S:2xNLS-YFP p35S:2xNLS-CFP* line#2<br>*p35S:2xNLS-YFP p35S:2xNLS-CFP* line#2<br>*p35S:2xNLS-YFP p35S:2xNLS-CFP* line#2	primary root tip<br>hypocotyl<br>stomata (mature leaf)	stable
Figure S13	*p35S:2xNLS-YFP p35S:2xNLS-CFP* line#1	primary root tip	stable
Figure S14	*p35S:2xNLS-YFP p35S:2xNLS-CFP* line#2	primary root tip	stable
Figure S15	*p35S:2xNLS-YFP p35S:2xNLS-CFP* line#1	hypocotyl	stable
Figure S16	*p35S:2xNLS-YFP p35S:2xNLS-CFP* line#2	hypocotyl	stable
Figure S17	*p35S:2xNLS-YFP p35S:2xNLS-CFP* line#1	stomata (mature leaf)	stable
Figure S18	*p35S:2xNLS-YFP p35S:2xNLS-CFP* line#2	stomata (mature leaf)	stable
Figure S19	*p35S:2xNLS-YFP p35S:2xNLS-CFP* line#1<br>*pUBQ10:2xNLS-YFP pUBQ10:2xNLS-CFP*	mature leaf	stable
Figure S20	*p35S:2xNLS-YFP p35S:2xNLS-CFP* line#2	mature leaf	stable
Figure S21	*p35S:2xNLS-YFP p35S:2xNLS-CFP* line#2	young leaf<br>mature leaf	stable
Figure S22	*p35S:2xNLS-YFP p35S:2xNLS-CFP* line#1	primary root tip<br>hypocotyl	stable
Figure S23	*p35S:2xNLS-YFP p35S:2xNLS-CFP* line#2	primary root tip<br>hypocotyl	stable
Figure S24	Simulation	-	-

Supplementary Table 2: Acquisition settings for fluorescent proteins.

Fluorescent protein	Laser line [nm] Excitation	Detection range [nm] Emmission
CFP	405	458-489
YFP	488	526-557
KikG	488	500-520
KikR	561	565-633

References

[1] M. Abramowitz and I. A. Stegun. *Handbook of Mathematical Functions: With Formulas, Graphs, and Mathematical Tables*. Dover Publications, 1964.

[2] C. G. Bowsher and P. S. Swain. Identifying sources of variation and the flow of information in biochemical networks. *Proceedings of the National Academy of Sciences*, 2012.

[3] M. B. Elowitz, A. J. Levine, E. D. Siggia, and P. S. Swain. Stochastic gene expression in a single cell. *Science (New York, NY)*, 2002.

[4] A. Hilfinger and J. Paulsson. Separating intrinsic from extrinsic fluctuations in dynamic biological systems. *Proceedings of the National Academy of Sciences*, 2011.

[5] S. Kalve, J. Fotschki, T. Beeckman, K. Vissenberg, and G. T. S. Beemster. Three-dimensional patterns of cell division and expansion throughout the development of Arabidopsis thaliana leaves. *Journal of Experimental Botany*, 2014.

[6] V. Shahrezaei and P. S. Swain. Analytical distributions for stochastic gene expression. *Proceedings of the National Academy of Sciences*, 2008.

[7] V. V. Verkhusha, I. M. Kuznetsova, O. V. Stepanenko, A. G. Zaraisky, M. M. Shavlovsky, K. K. Turoverov, and V. N. Uversky. High Stability of Discosoma DsRed As Compared to Aequorea EGFP. *Biochemistry*, 2003.

Supplementary Fig. 1

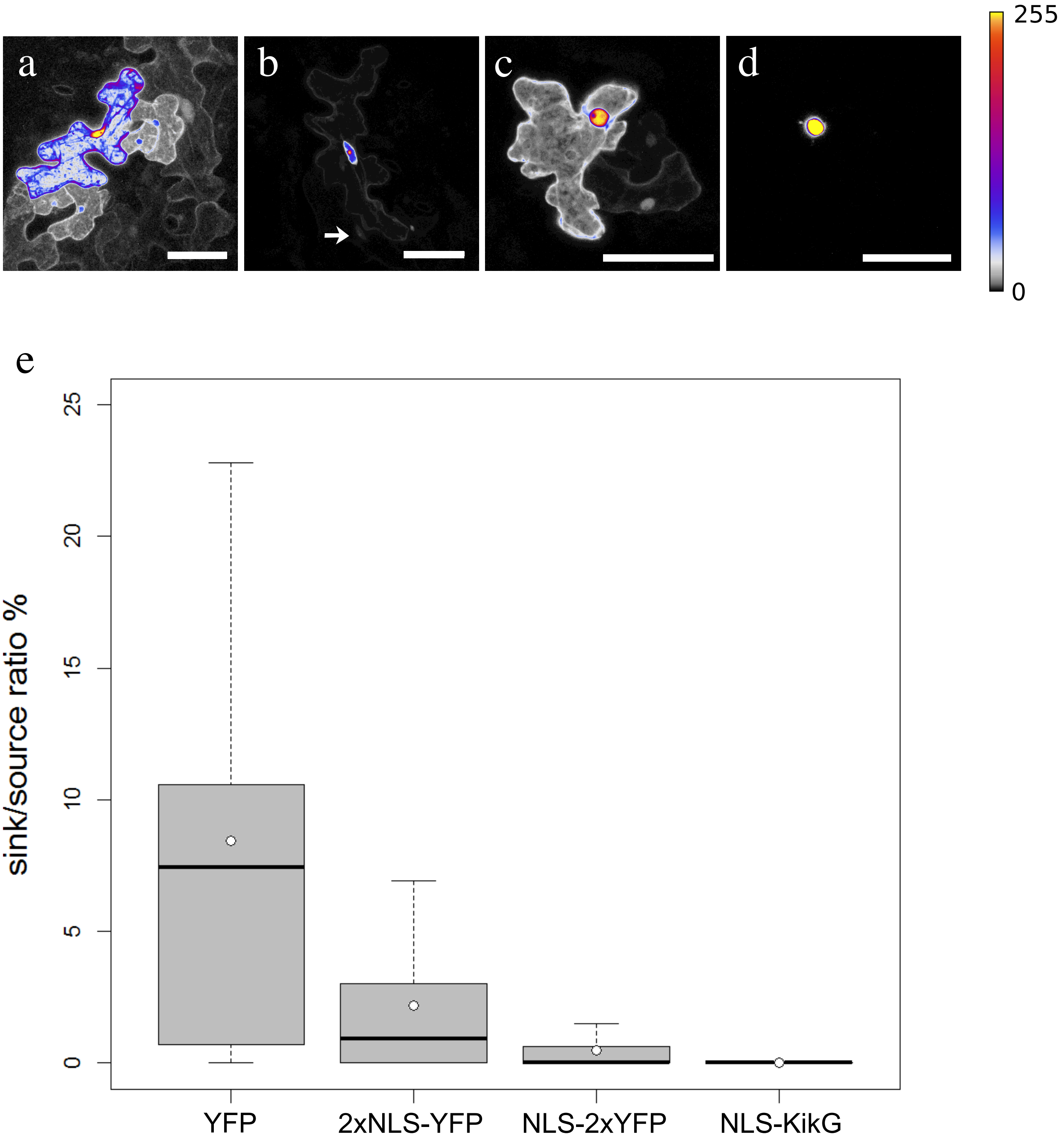

Supplementary Fig. 1: Comparison of protein localisation and movement of YFP variants

(A-D) Representative CLSM images of transiently transformed single pavement cells in Arabidopsis expressing YFP-tagged and KikG marker proteins and marker movement into neighbouring cells. The colour code indicates the signal intensity in a scale between 0 and 255. (A) Free YFP. (B) 2xNLS-YFP. Arrow indicates fluorescence in the neighbouring cell. (C) NLS-2xYFP. (D) NLS-KikG. Colour code legend indicates grey scale values from 0 to 255 in A-D. (E) Box plot diagram of the ratio between sink and source cells in %. This corresponds to the mobility coefficient ε (see supplement). YFP (n=48, $\varepsilon = 0.0843$), 2xNLS-YFP (n=65, $\varepsilon = 0.0218$), NLS-2xYFP (n=61, $\varepsilon = 0.0046$) and NLS-KikG (n=20, $\varepsilon = 0.0$). Ratios for 2xNLS-YFP were significantly lower compared to YFP (Kolmogorov Smirnov test $p < 3 \times 10^{-6}$). Ratios for NLS-2xYFP was significantly reduced compared to 2xNLS-YFP and free YFP (Kolmogorov Smirnov test $p < 2 \times 10^{-4}$ and $p < 6 \times 10^{-12}$). Boxes show 25th, 75th quartiles and median. White dots show mean values.

Supplementary Fig. 2

a

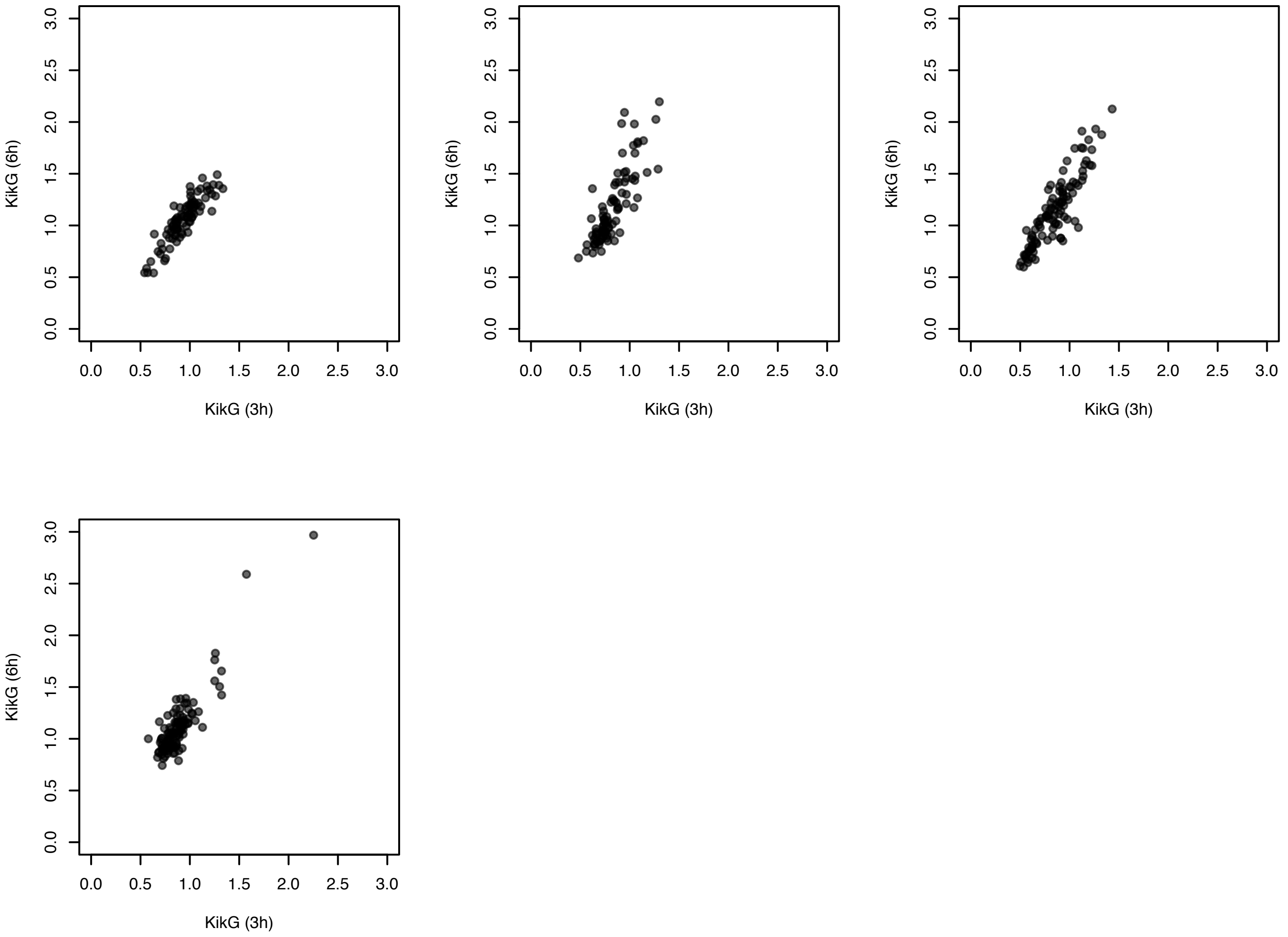

b

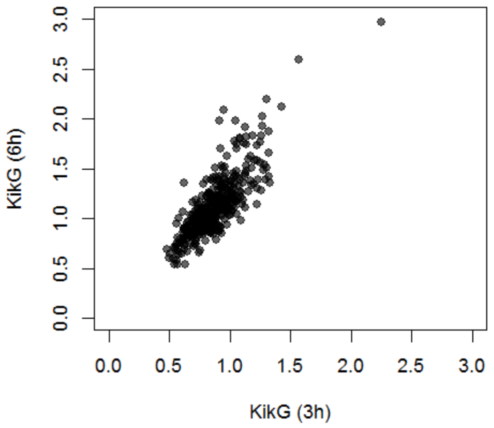

Supplementary Fig. 2: Scatter plots of *p35S:NLS-KikG*expressing cells.

A) Scatter plot of *p35S:NLS-KikG* expressing cells obtained from four individual leaves. B) Cumulative scatter plot of all leaves.

Supplementary Fig. 3

a

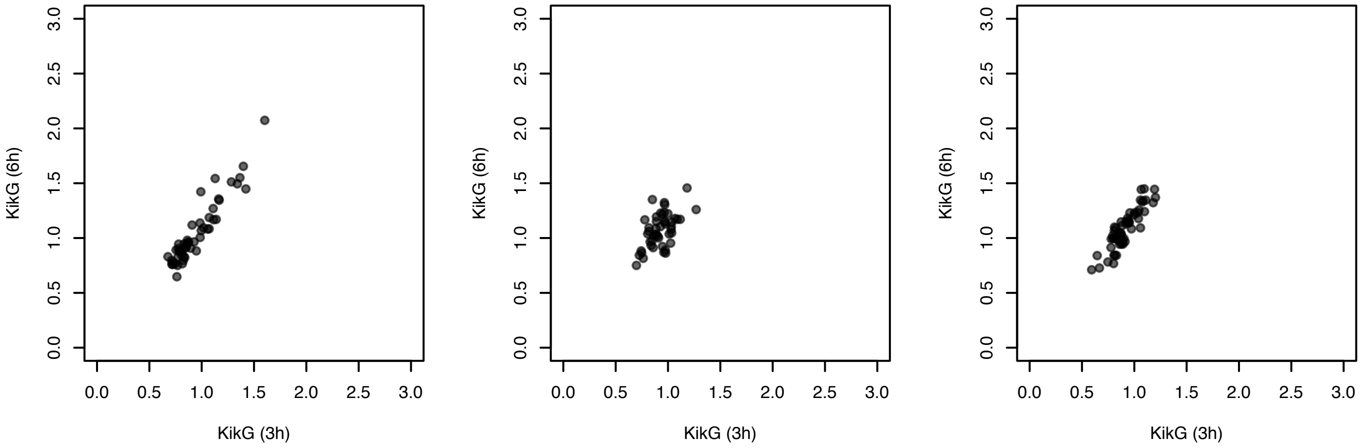

b

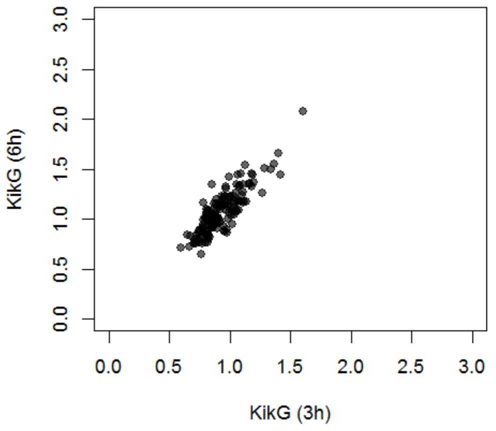

Supplementary Fig. 3: Scatter plots of *pUBQ10:NLS-KikG* expressing cells.

A) Scatter plot of *pUBQ10:NLS-KikG* expressing cells obtained from three individual leaves. B) Cumulative scatter plot of all leaves.

Supplementary Fig. 4

a

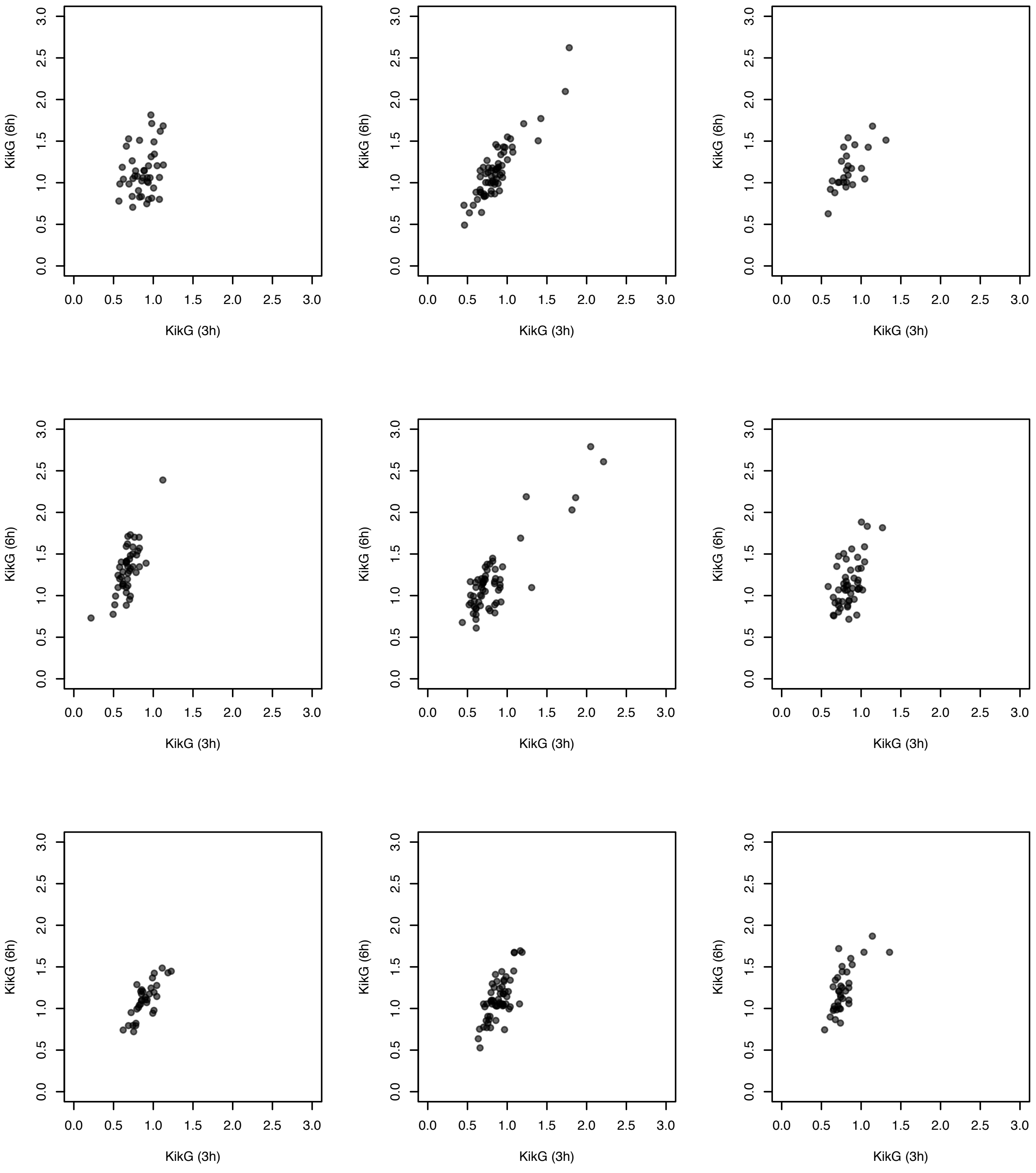

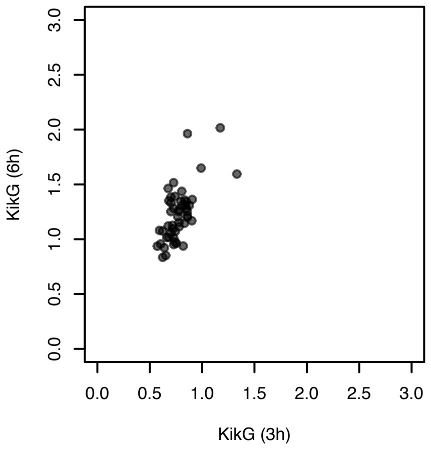

b

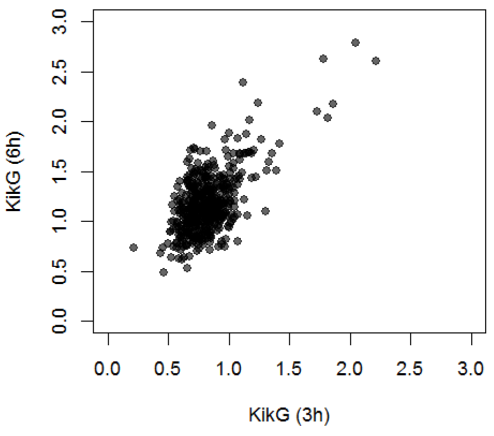

Supplementary Fig. 4: Scatter plots of *p35S:NLS-KikG* expressing cells without dark treatment.

A) Scatter plot of *p35S:NLS-KikG* expressing cells obtained from ten individual leaves. B) Cumulative scatter plot of all leaves.

Supplementary Fig. 5

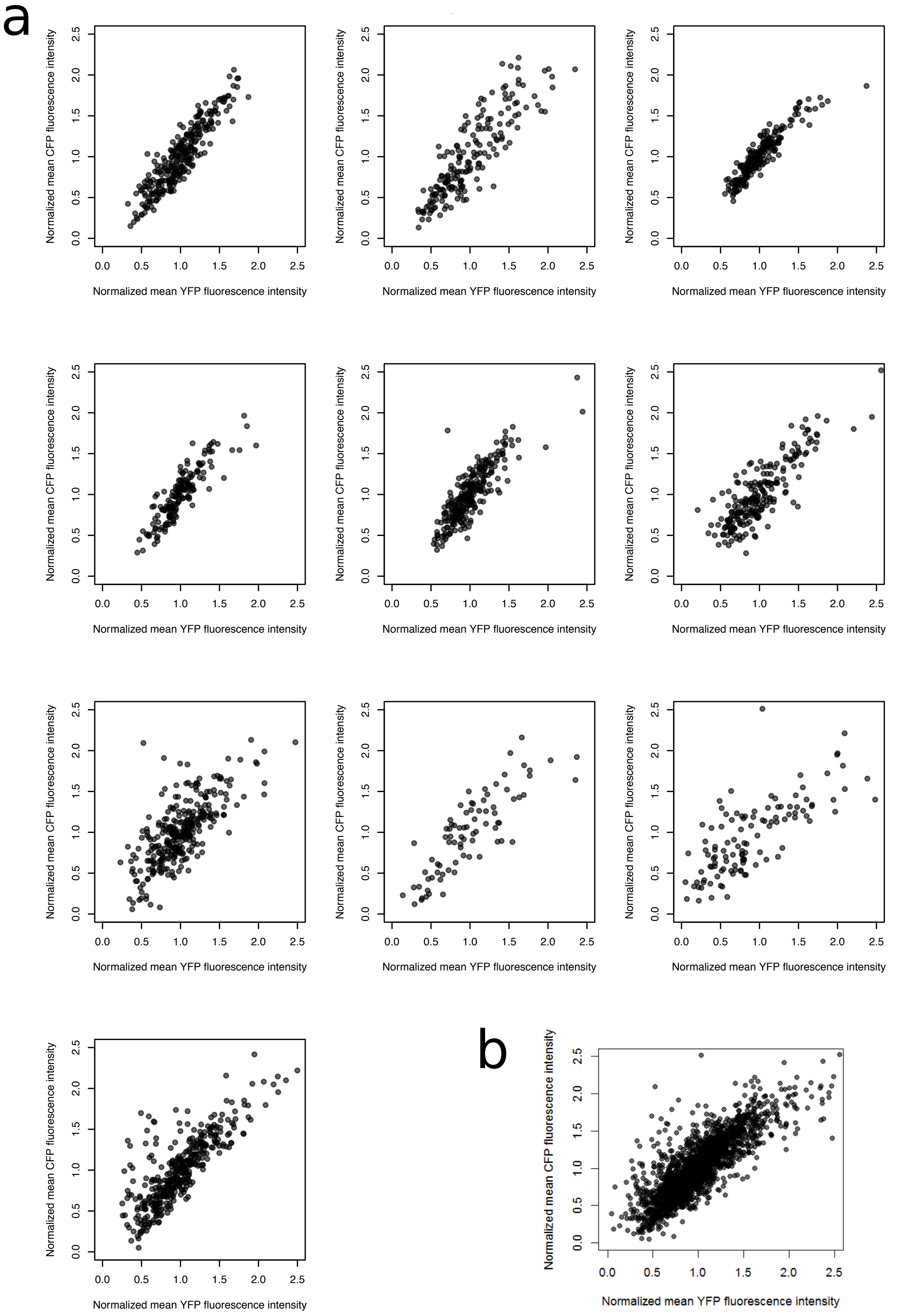

Supplementary Fig. 5: Scatter plots of the CFP and YFP values in young leaves of *p35S:2xNLS-YFP p35S:2xNLS-CFP* plants (Transgenic line 1).

(A) Scatter plots of the CFP and YFP values obtained from ten individual young leaves. A Kolmogorov Smirnov Test was used to test, whether the CFP and YFP value distribution significantly differed. This was not the case in all samples used in this study. (B) Cumulative scatter plot combining all samples.

Supplementary Fig. 6

a

b

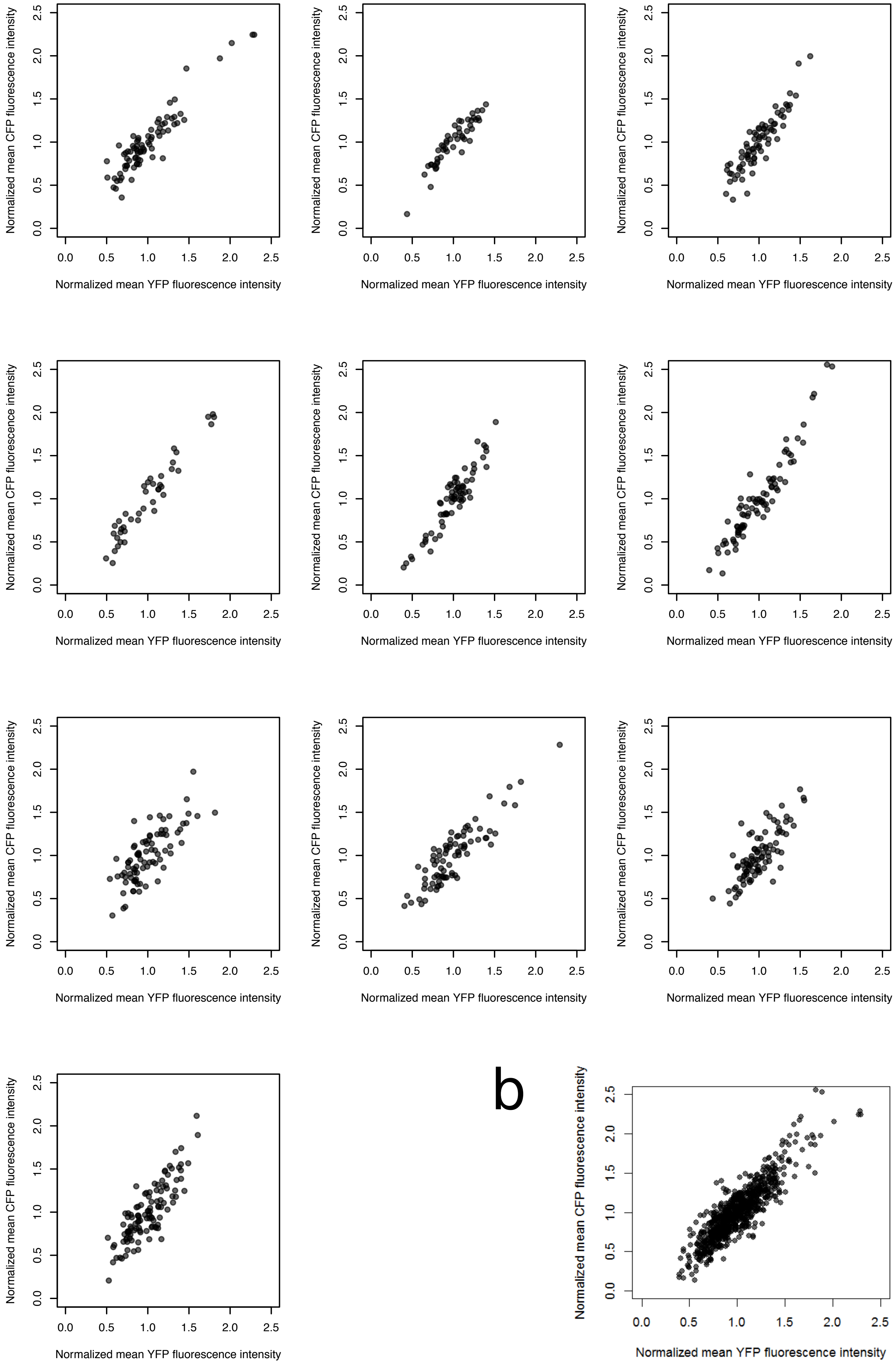

Supplementary Fig. 6: Scatter plots of the CFP and YFP values in mature leaves of *p35S:2xNLS-YFP p35S:2xNLS-CFP* plants (Transgenic line 1).

(A) Scatter plots of the CFP and YFP values obtained from ten individual mature leaves. A Kolmogorov Smirnov Test was used to test, whether the CFP and YFP value distribution significantly differed. This was not the case in all samples used in this study. (B) Cumulative scatter plot combining all samples.

Supplementary Fig. 7

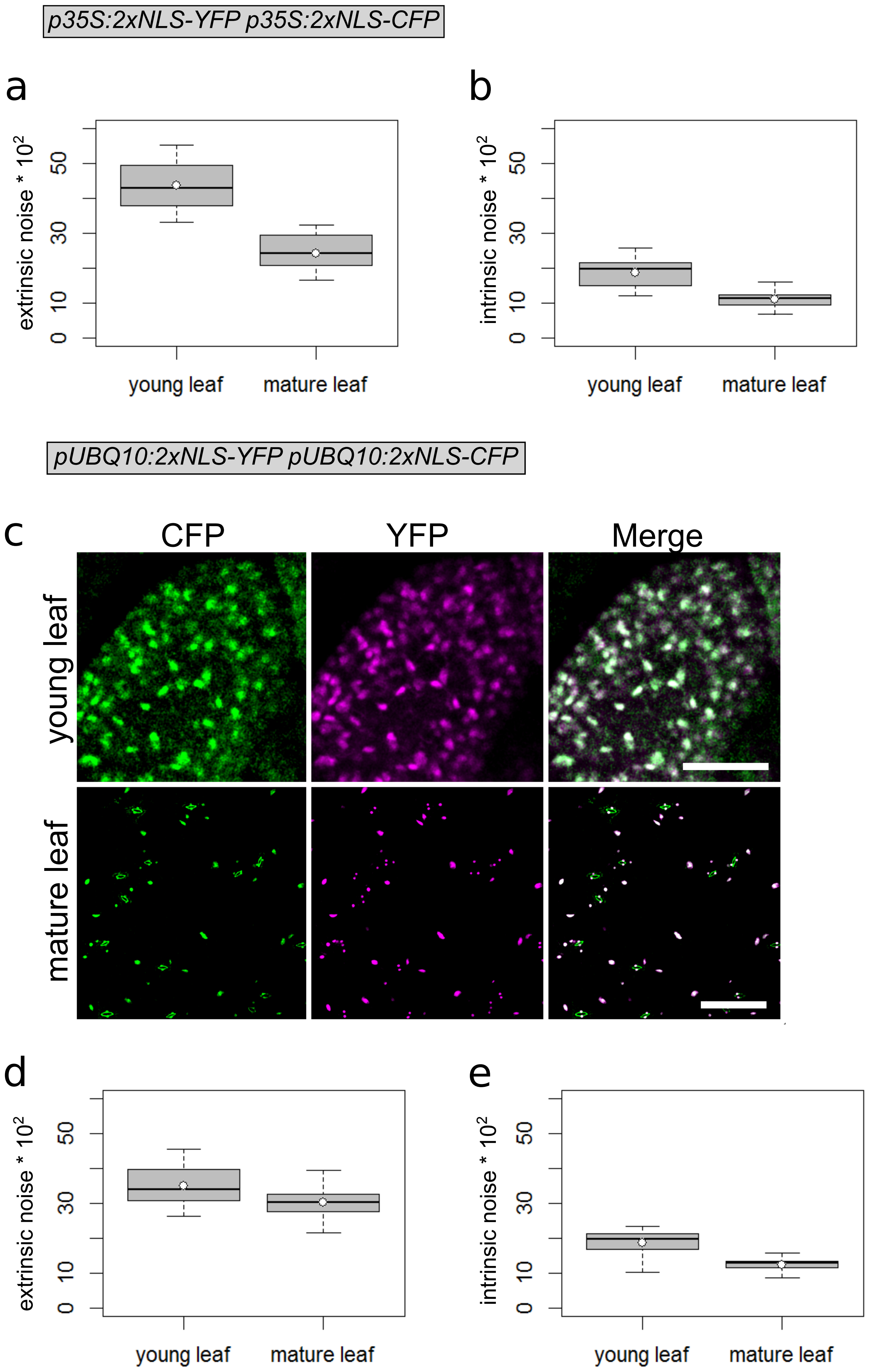

Supplementary Fig. 7: Intrinsic and extrinsic noise in young and mature rosette leaves of a second independently transformed *p35S:2xNLS-YFP p35S:2xNLS-CFP* line (Transgenic line 2) and *pUBQ10:2xNLS-YFP pUBQ10:2xNLS-CFP* plants.

(A) Box plot of extrinsic noise measurements of young leaves (7 leaves with a total number of 1020 cells, median=42.9) and mature leaves (9 leaves with a total number of 796 cells, median=24.0). The extrinsic noise in young leaves was significantly higher compared to mature leaves ($p = 0.0002$, Wilcoxon rank sum test). Boxes show 25th and 75th quartiles and median. White dots show mean values. (B) Box plot of intrinsic noise of young (7 leaves with a total number of 1020 cells, median=19.7) and mature leaves (9 leaves with a total number of 796 cells, median=11.3). The intrinsic noise in young leaves was significantly higher compared to mature leaves ($p = 0.005$, Wilcoxon rank sum test). The extrinsic noise in mature and young leaves was significantly higher than the intrinsic noise ($p = 4.1 \times 10^{-5}$ and $p = 0.0006$, Wilcoxon rank-sum test). (C) CLSM image of a young and a mature leaf of *pUBQ10:2xNLS-YFP pUBQ10:2xNLS-CFP*. YFP is shown in magenta, CFP in green, overlapping fluorescence intensities are white. Note the auto-fluorescence in the CFP channel of stomata. Scale bar: 30 µm (upper row) and 100 µm (lower row). (D) Box plot of extrinsic noise measurements of young (8 leaves with a total number of 2021 cells, median=33.9) and mature leaves (12 leaves with a total number of 775 cells, median=30.3). The extrinsic noise in young leaves was not significantly higher compared to mature leaves ($p = 0.082$, Wilcoxon rank sum test). (E) Box plot of intrinsic noise measurements of young (8 leaves with a total number of 2120 cells, median=19.6) and mature leaves (12 leaves with a total number of 775 cells, median=12.8). The intrinsic noise in young leaves was significantly higher compared to mature leaves ($p = 0.003$, Wilcoxon rank sum test). The extrinsic noise of young and mature leaves was significantly higher compared to the intrinsic noise ($p = 7.4 \times 10^{-7}$ and $p = 0.0002$, Wilcoxon signed-rank test).

Supplementary Fig. 8

a

b

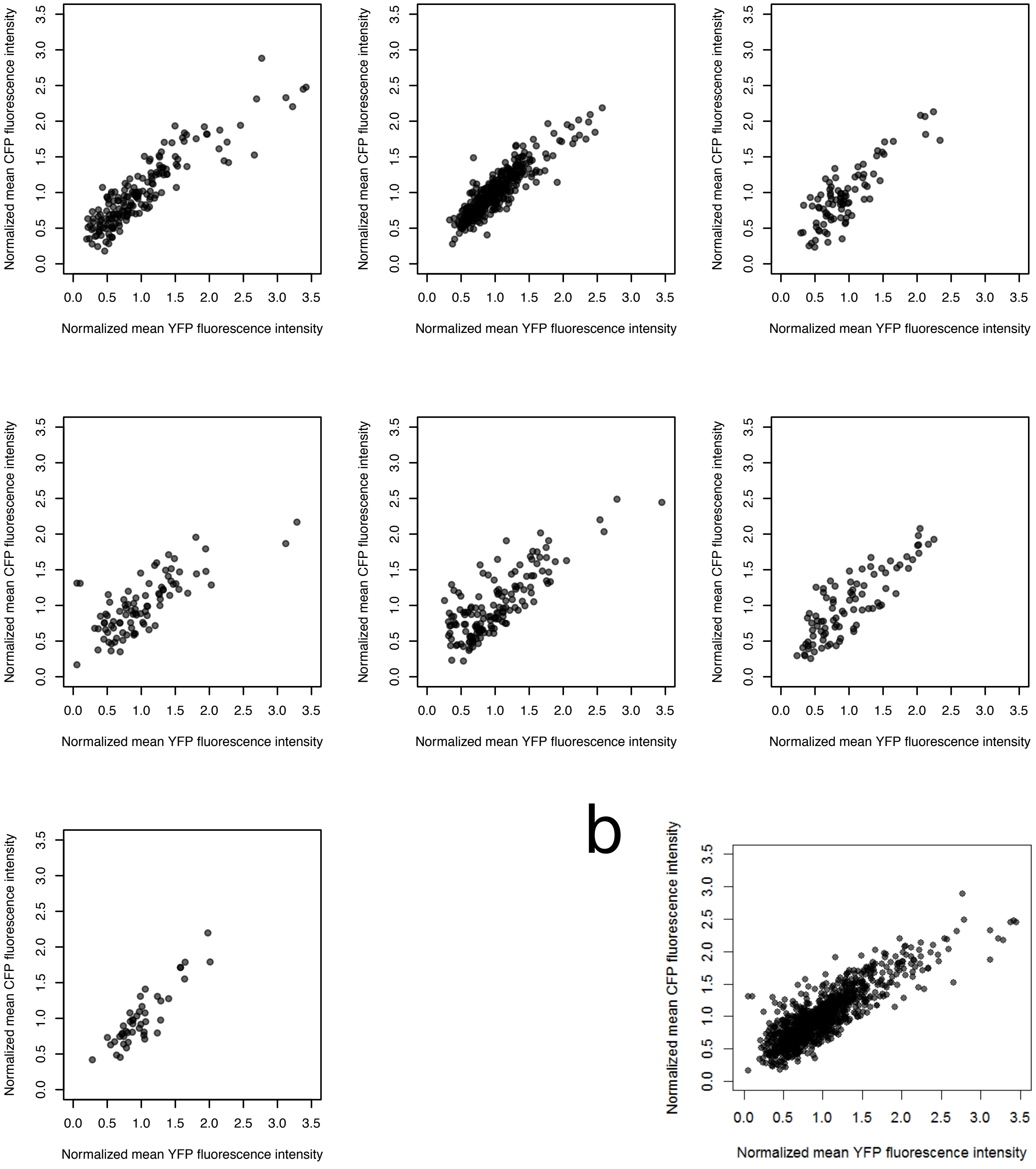

Supplementary Fig. 8: Scatter plots of the CFP and YFP values in young leaves of *p35S:2xNLS-YFP p35S:2xNLS-CFP* plants (Transgenic line 2).

(A) Scatter plots of the CFP and YFP values obtained from seven individual young leaves. A Kolmogorov Smirnov Test was used to test, whether the CFP and YFP value distribution significantly differed. This was not the case in all samples used in this study. (B) Cumulative scatter plot combining all samples.

Supplementary Fig. 9

a

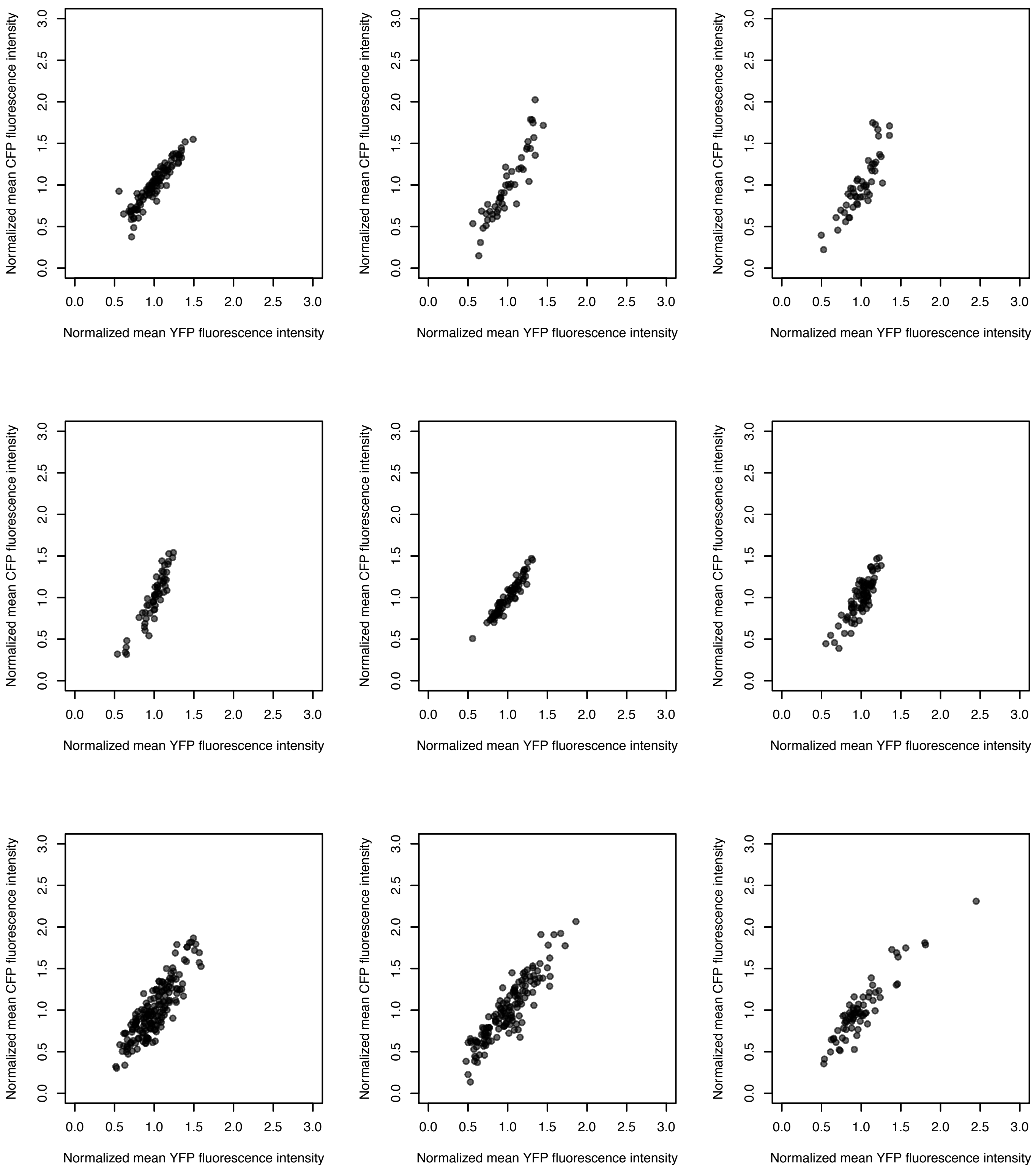

b

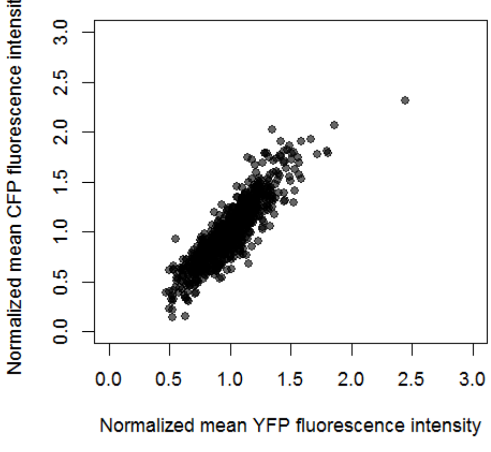

Supplementary Fig. 9: Scatter plots of the CFP and YFP values in mature leaves of *p35S:2xNLS-YFP p35S:2xNLS-CFP* plants (Transgenic line 2).

(A) Scatter plots of the CFP and YFP values obtained from nine individual mature leaves. A Kolmogorov Smirnov Test was used to test, whether the CFP and YFP value distribution significantly differed. This was not the case in all samples used in this study. (B) Cumulative scatter plot combining all samples.

Supplementary Fig. 10

a

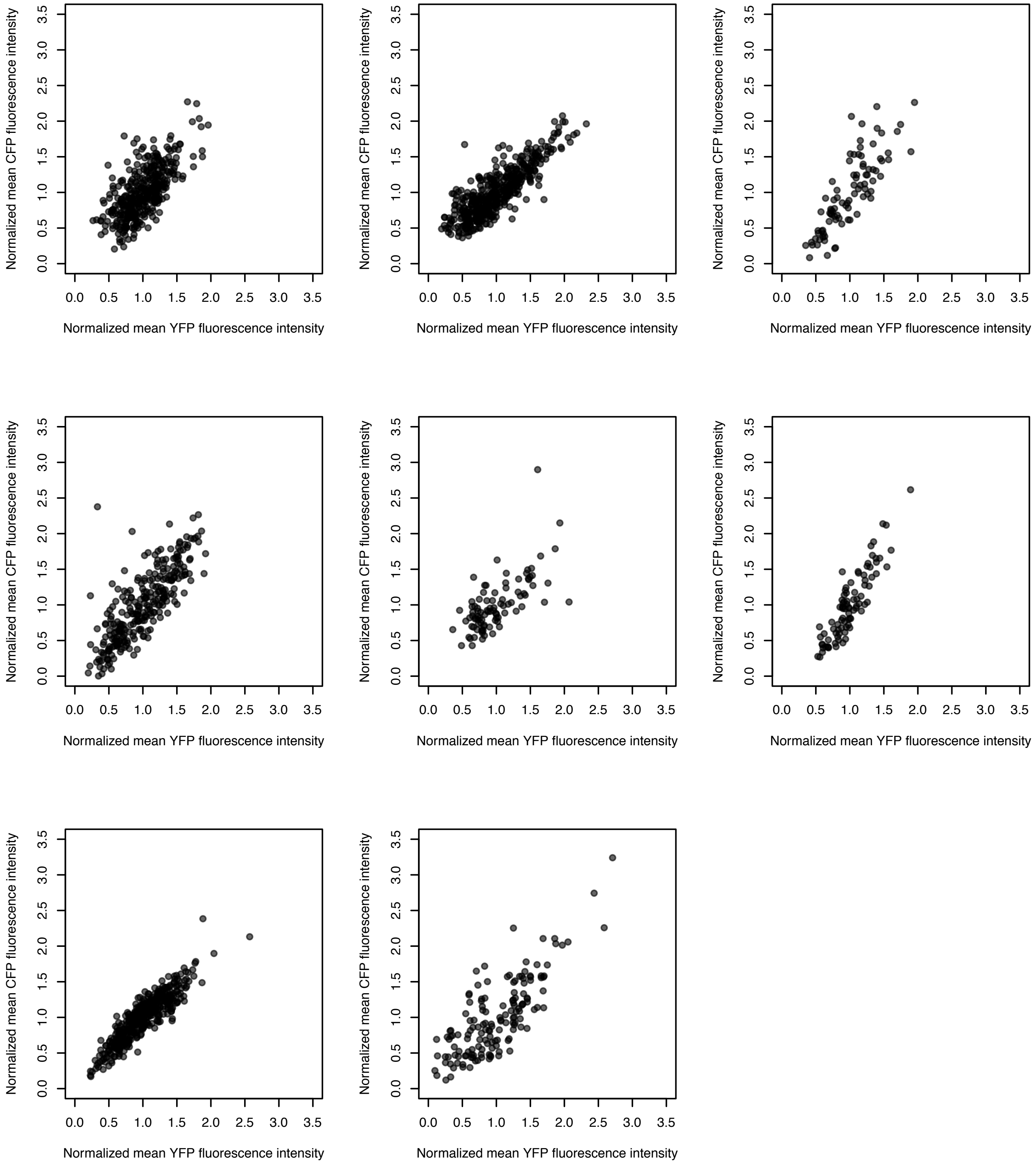

b

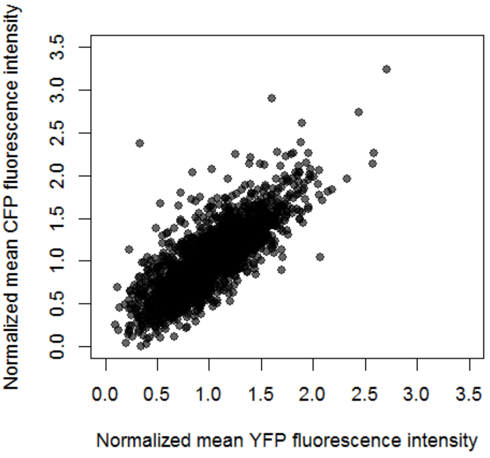

Supplementary Fig. 10: Scatter plots of the CFP and YFP values in young leaves *of pUBQ10:2xNLS-YFP pUBQ10:2xNLS-CFP* plants.

(A) Scatter plots of the CFP and YFP values obtained from eight individual young leaves. A Kolmogorov Smirnov Test was used to test, whether the CFP and YFP value distribution significantly differed. This was not the case in all samples used in this study. (B) Cumulative scatter plot combining all samples.

Supplementary Fig. 11

a

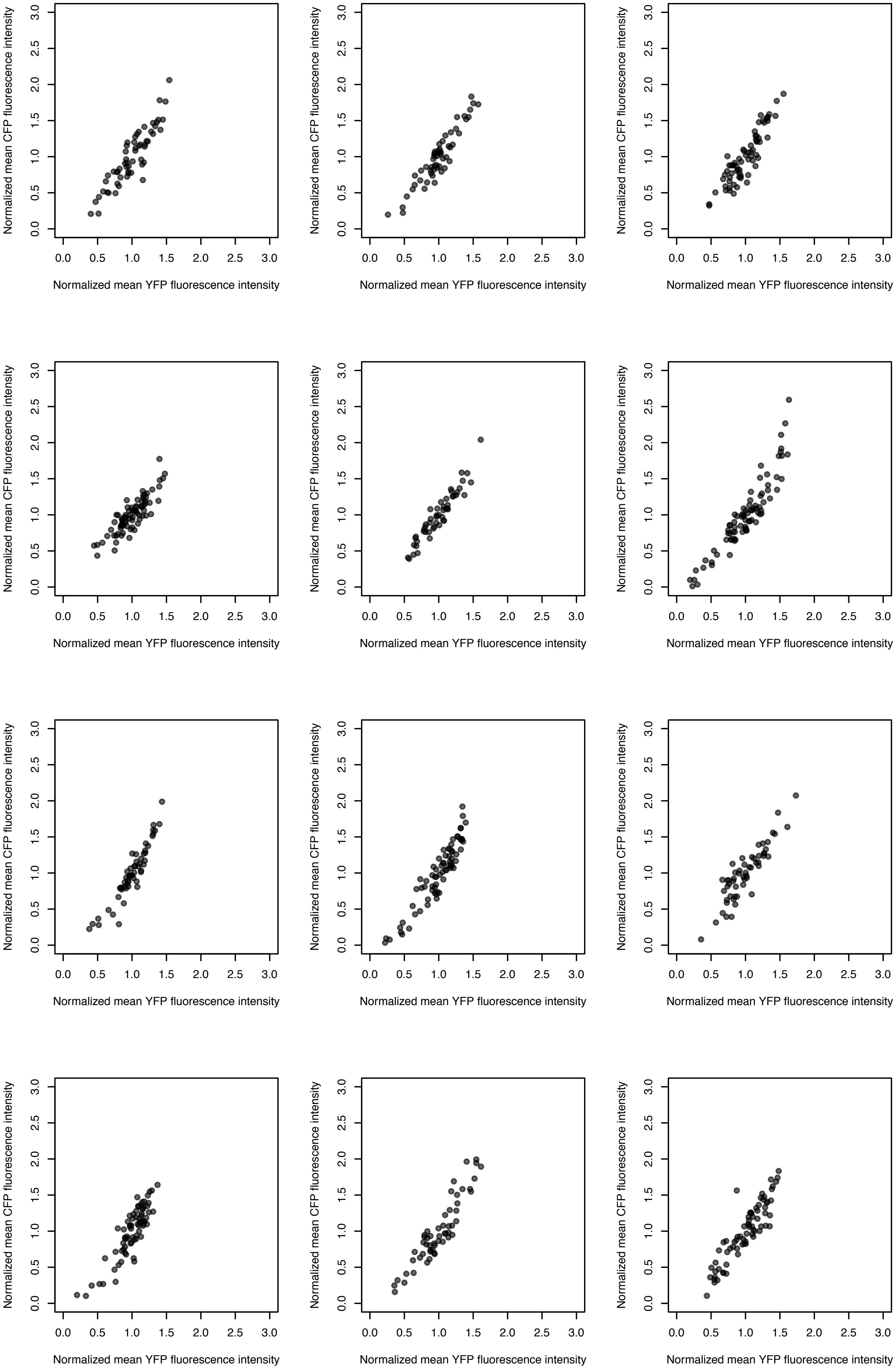

b

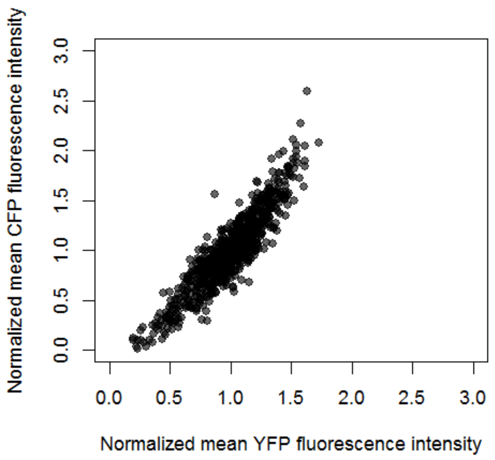
Normalized mean CFP fluorescence intensity
0.0
0.5
1.0
1.5
2.0
2.5
3.0
0.0
0.5
1.0
1.5
2.0
2.5
3.0
Normalized mean YFP fluorescence intensity

Supplementary Fig. 11: Scatter plots of the CFP and YFP values in mature leaves of *pUBQ10:2xNLS-YFP pUBQ10:2xNLS-CFP* plants.

(A) Scatter plots of the CFP and YFP values obtained from twelve individual mature leaves. A Kolmogorov Smirnov Test was used to test, whether the CFP and YFP value distribution significantly differed. This was not the case in all samples used in this study. (B) Cumulative scatter plot combining all samples.

Supplementary Fig. 12

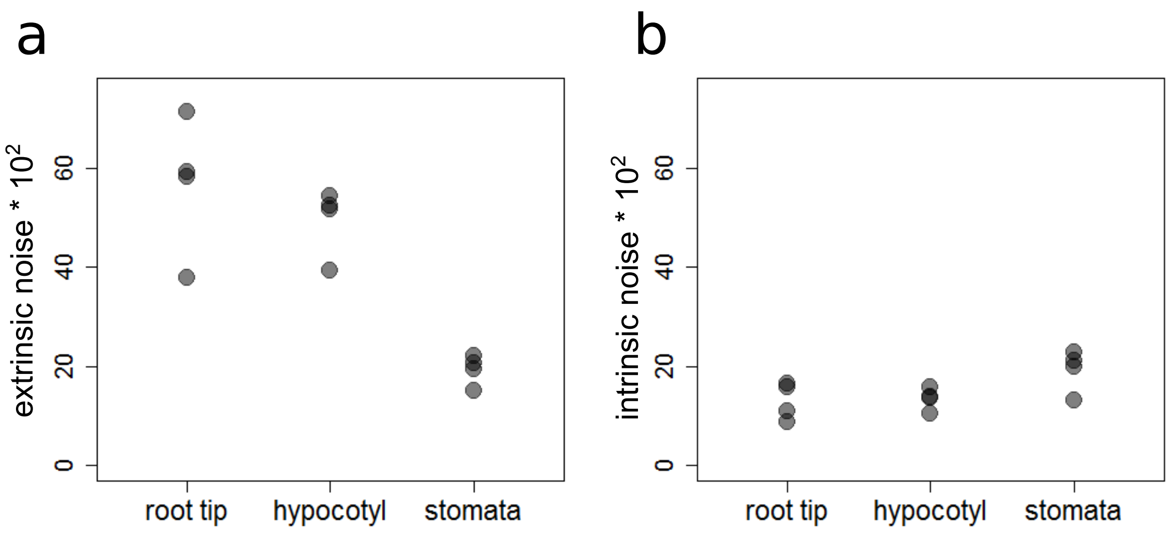

Supplementary Fig. 12: Extrinsic and intrinsic noise in epidermal stomata cells, root tip cells and hypocotyls cells of a second independently transformed *p35S:2xNLS-YFP p35S:2xNLS-CFP* line (Transgenic line 2).
(A) Plot of extrinsic noise of root tip cells (n=4 root tips with a total number of 385 cells, median=58.7), hypocotyls cells (n=4 hypocotyls with a total number of 481 cells, median=52.0) and stomata cells (n=4 mature leaves with a total number of 184 stomata cells, median=20.1). (B) Plot of intrinsic noise of root tip cells (n=4 root tips with a total number of 385 cells, median=13.4), hypocotyls cells (n=4 hypocotyls with a total number of 481 cells, median=13.7) and stomata cells (n=4 mature leaves with a total number of 184 stomata cells, median=20.6). The extrinsic noise in root tip cells and hypocotyls cells was significantly higher as in stomata cells ($p = 0.029$ and $p = 0.029$, Wilcoxon rank-sum test).

Supplementary Fig. 13

a

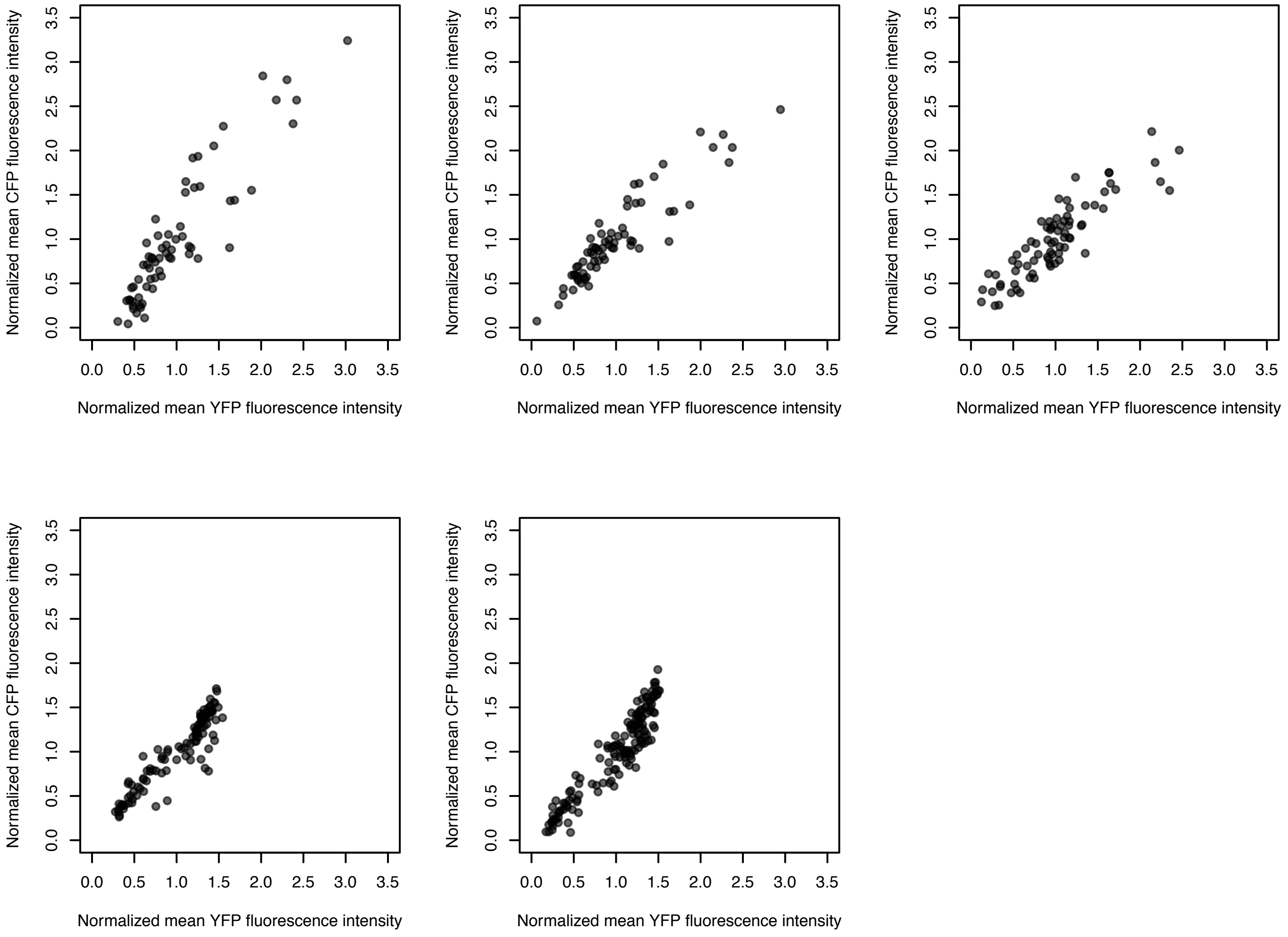

b

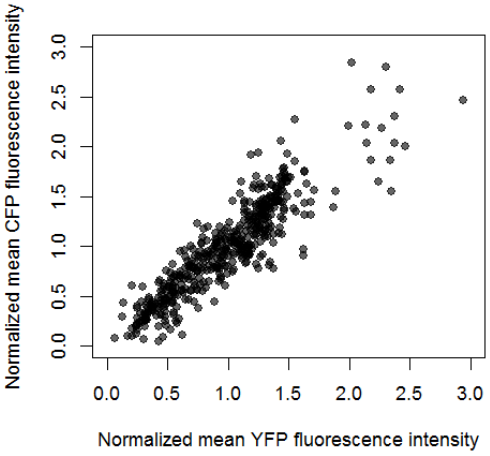

Supplementary Fig. 13: Scatter plots of the CFP and YFP values in root tips of *p35S:2xNLS-YFP p35S:2xNLS-CFP* plants (Transgenic Line 1).

(A) Scatter plots of the CFP and YFP values obtained from five individual root tips. A Kolmogorov Smirnov Test was used to test, whether the CFP and YFP value distribution significantly differed. This was not the case in all samples used in this study. (B) Cumulative scatter plot combining all samples.

Supplementary Fig. 14

a

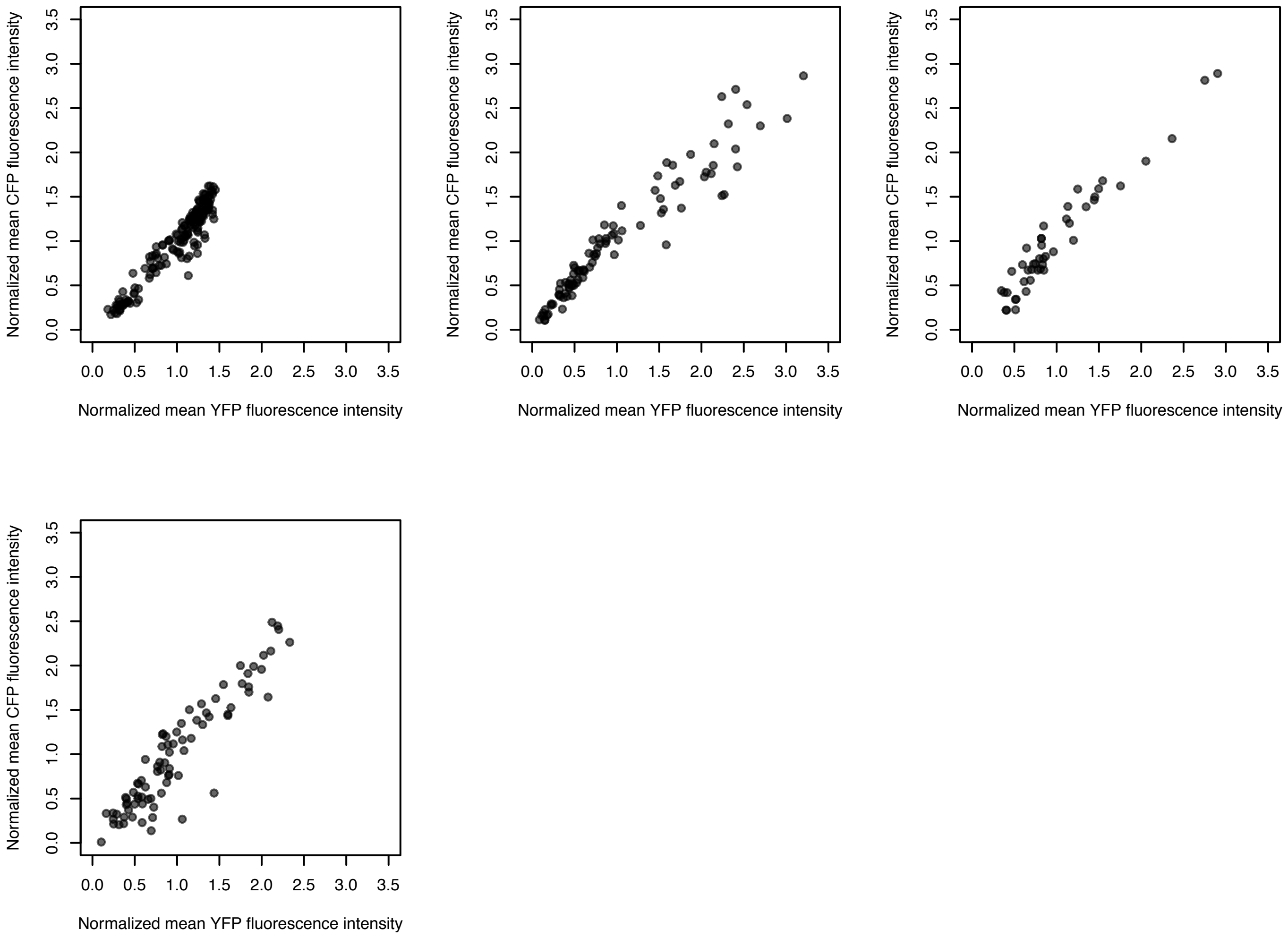

b

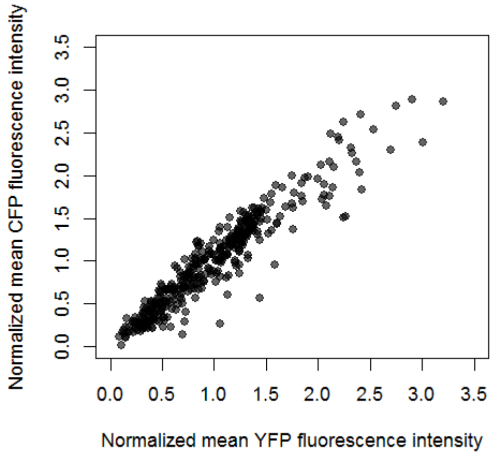

Supplementary Fig. 14: Scatter plots of the CFP and YFP values in root tips of *p35S:2xNLS-YFP p35S:2xNLS-CFP* plants (Transgenic Line 2).

(A) Scatter plots of the CFP and YFP values obtained from four individual root tips. A Kolmogorov Smirnov Test was used to test, whether the CFP and YFP value distribution significantly differed. This was not the case in all samples used in this study. (B) Cumulative scatter plot combining all samples.

Supplementary Fig. 15

a

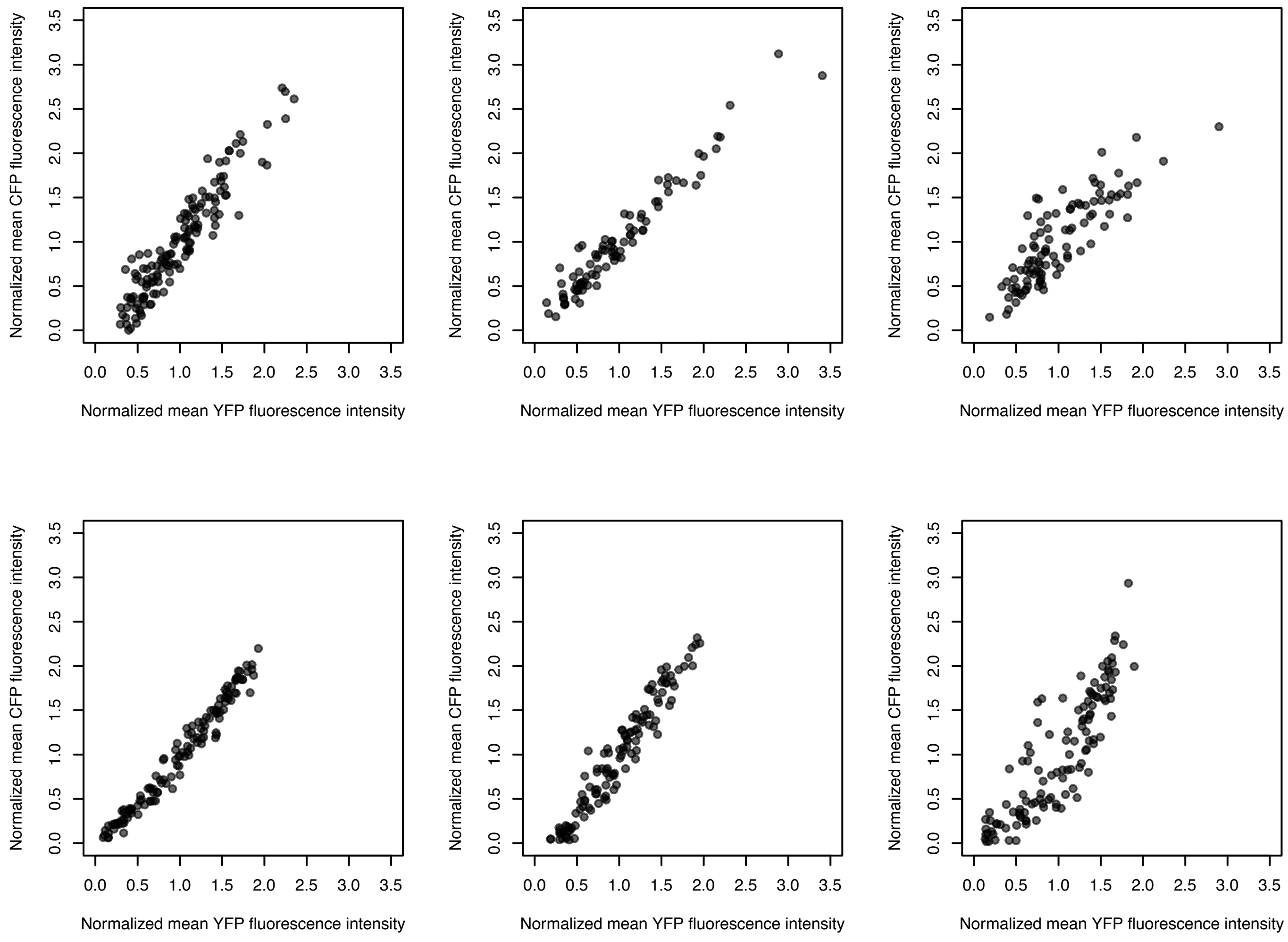

b

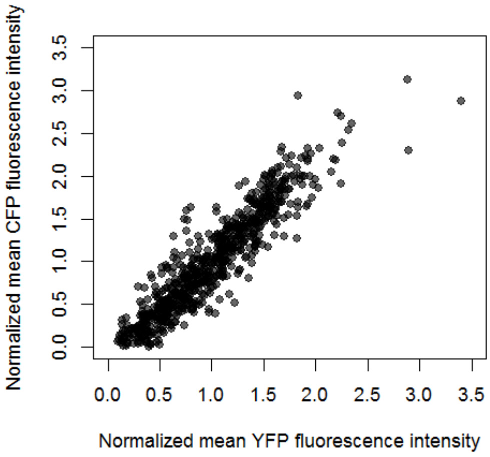

Supplementary Fig. 15: Scatter plots of the CFP and YFP values in hypocotyl of *p35S:2xNLS-YFP p35S:2xNLS-CFP* plants (Transgenic Line 1).

(A) Scatter plots of the CFP and YFP values obtained from six individual hypocotyls. A Kolmogorov Smirnov Test was used to test, whether the CFP and YFP value distribution significantly differed. This was not the case in all samples used in this study. (B) Cumulative scatter plot combining all samples.

Supplementary Fig. 16

a

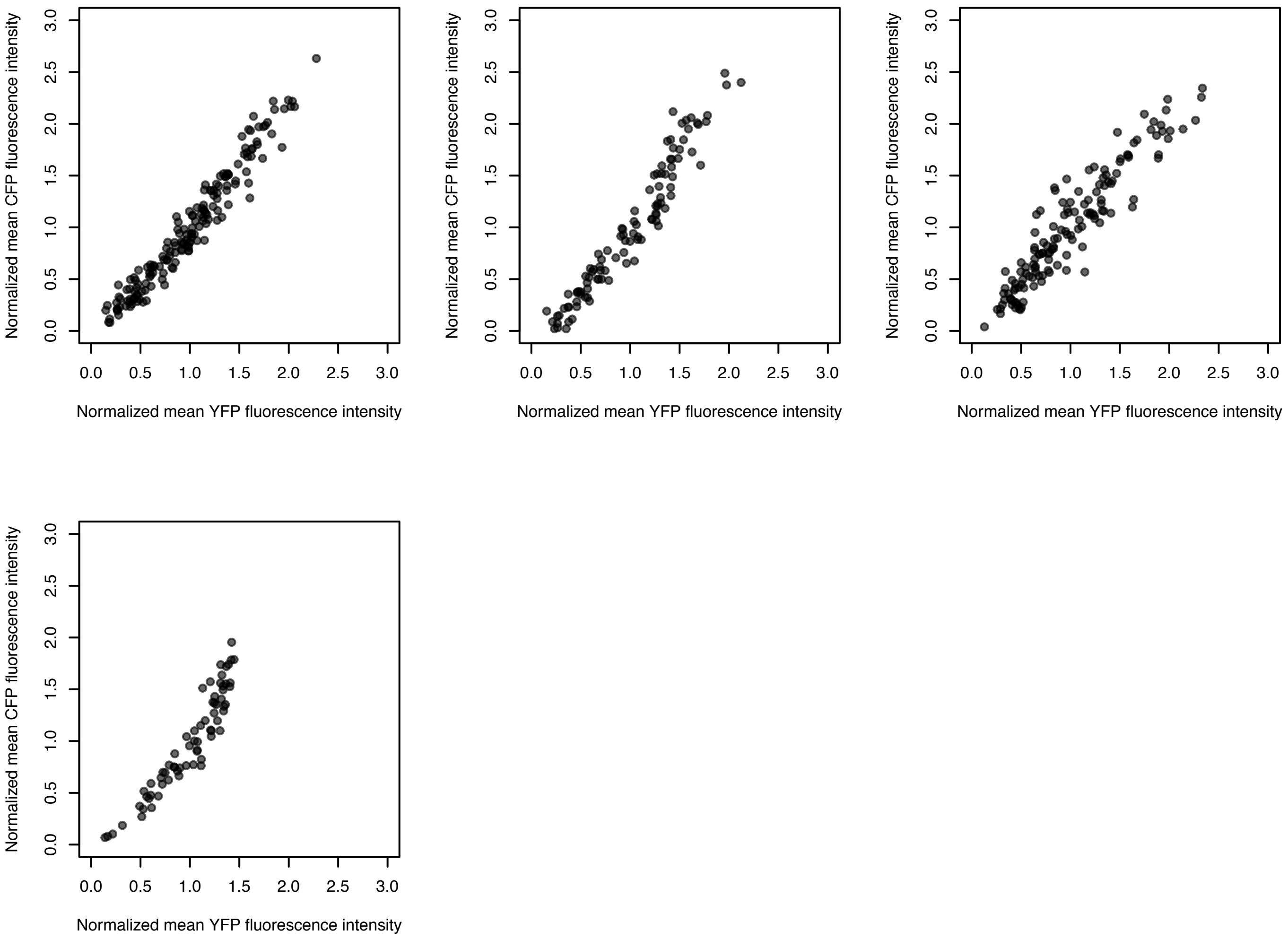

b

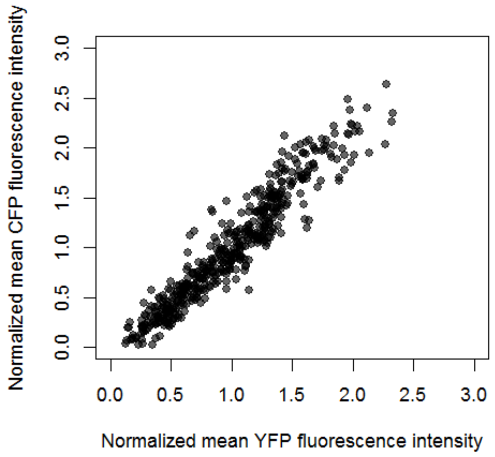

Supplementary Fig. 16: Scatter plots of the CFP and YFP values in hypocotyl cells of *p35S:2xNLS-YFP p35S:2xNLS-CFP* plants (Transgenic Line 2).

(A) Scatter plots of the CFP and YFP values obtained from four individual hypocotyls. A Kolmogorov Smirnov Test was used to test, whether the CFP and YFP value distribution significantly differed. This was not the case in all samples used in this study. (B) Cumulative scatter plot combining all samples.

Supplementary Fig. 17

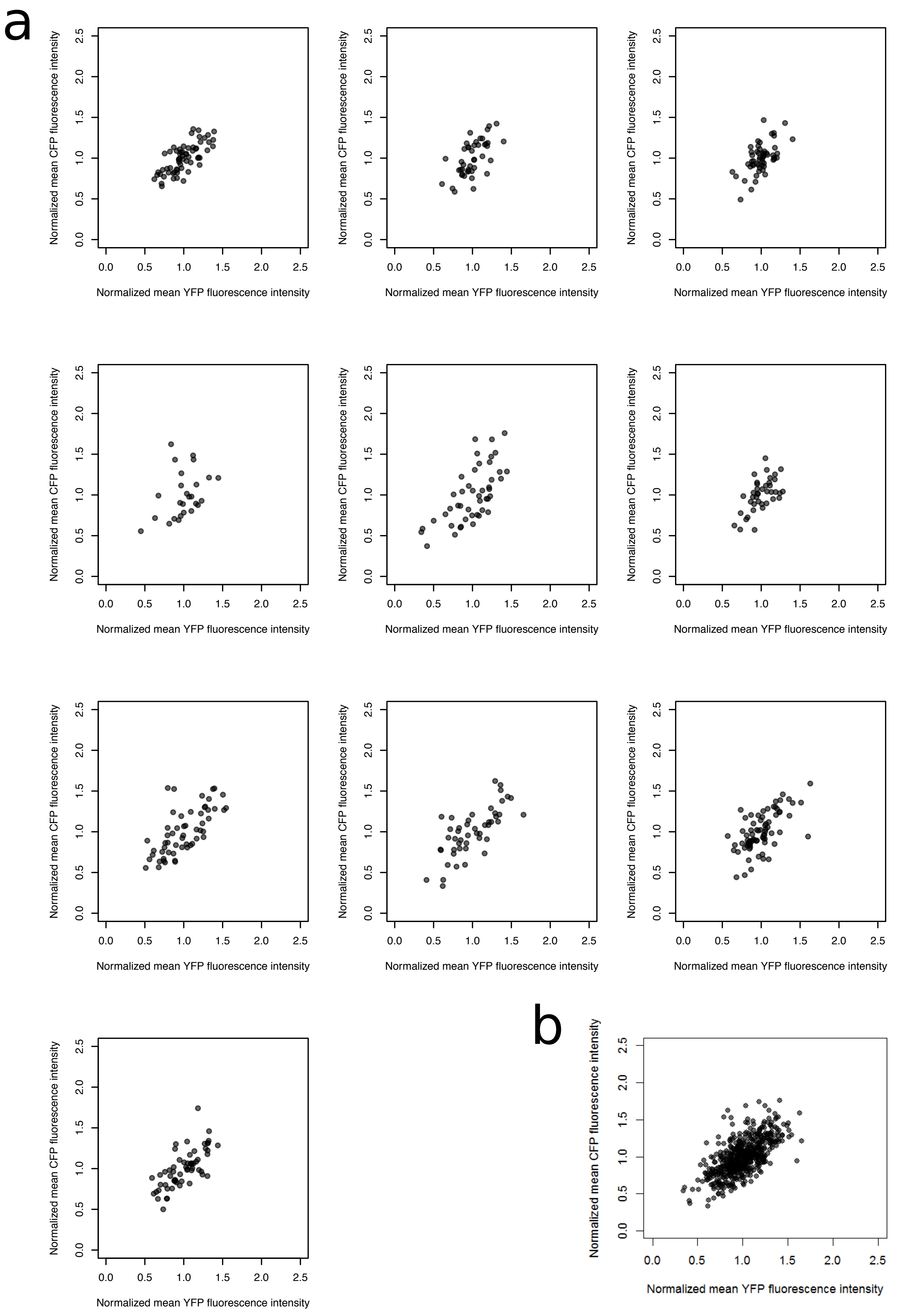

Supplementary Fig. 17: Scatter plots of the CFP and YFP values in stomata of *p35S:2xNLS-YFP p35S:2xNLS-CFP* plants (Transgenic Line 1).

(A) Scatter plots of the CFP and YFP values obtained from stomata on ten individual mature leaves. A Kolmogorov Smirnov Test was used to test, whether the CFP and YFP value distribution significantly differed. This was not the case in all samples used in this study. (B) Cumulative scatter plot combining all samples.

Supplementary Fig. 18

a

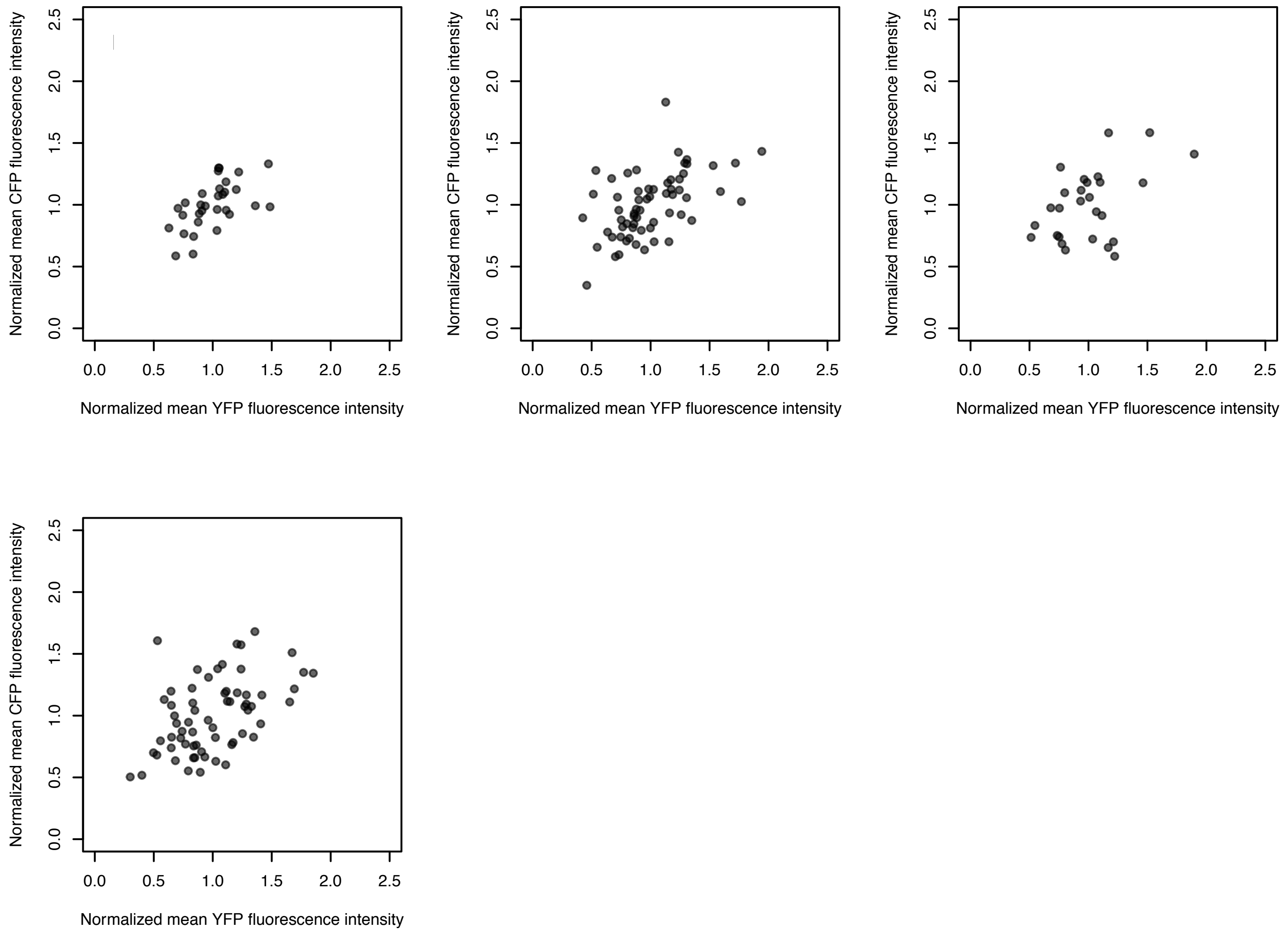

b

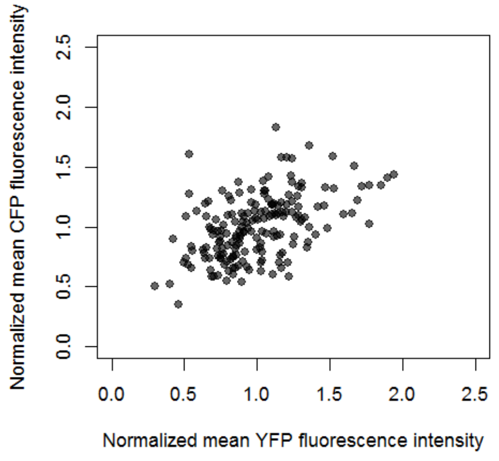

Supplementary Fig. 18: Scatter plots of the CFP and YFP values in stomata of *p35S:2xNLS-YFP p35S:2xNLS-CFP* plants (Transgenic Line 2).

(A) Scatter plots of the CFP and YFP values obtained from stomata on four individual mature leaves. A Kolmogorov Smirnov Test was used to test, whether the CFP and YFP value distribution significantly differed. This was not the case in all samples used in this study. (B) Cumulative scatter plot combining all samples.

Supplementary Fig. 19

p35S:2xNLS-YFP p35S:2xNLS-CFP

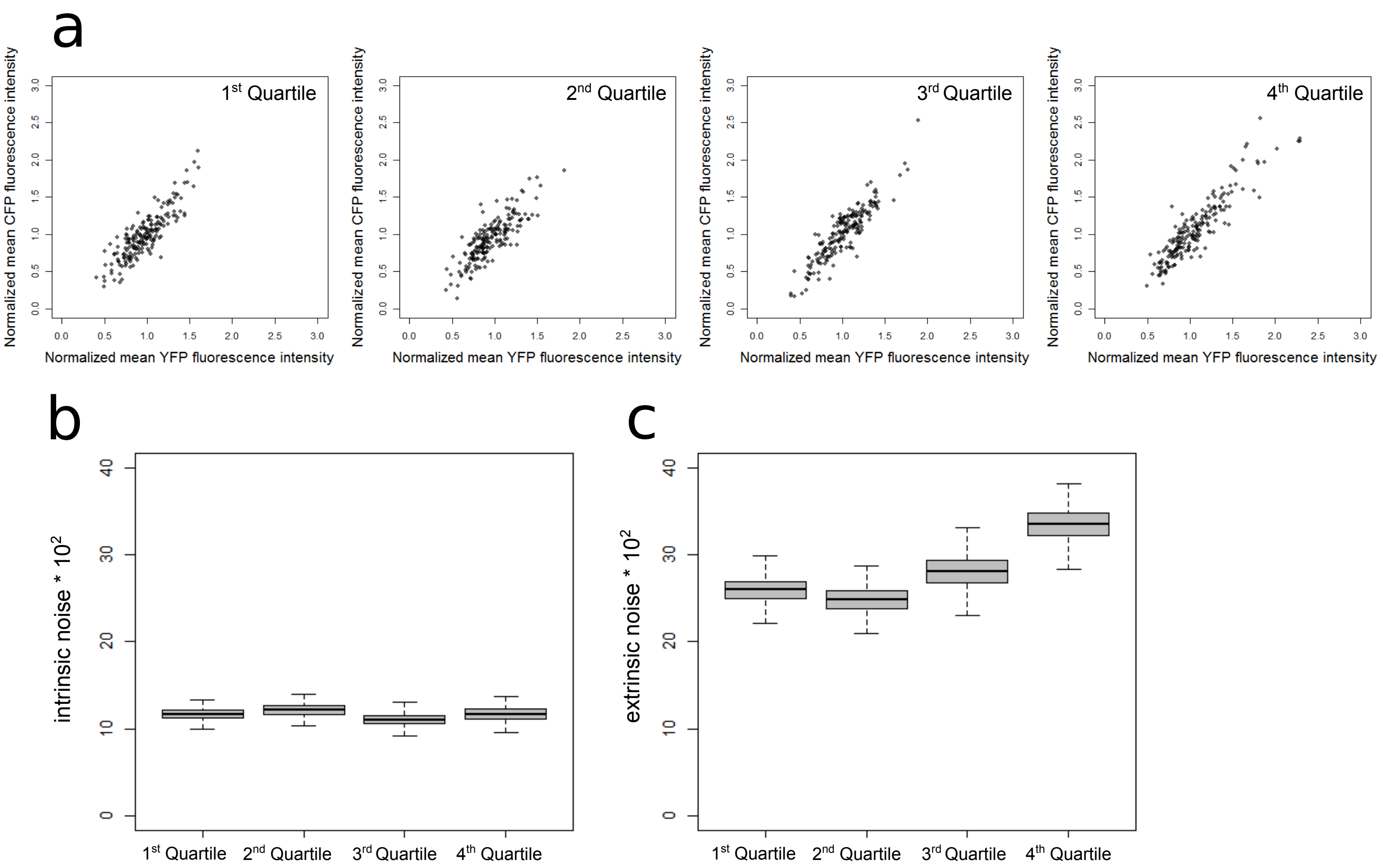

pUBQ10:2xNLS-YFP pUBQ10:2xNLS-CFP

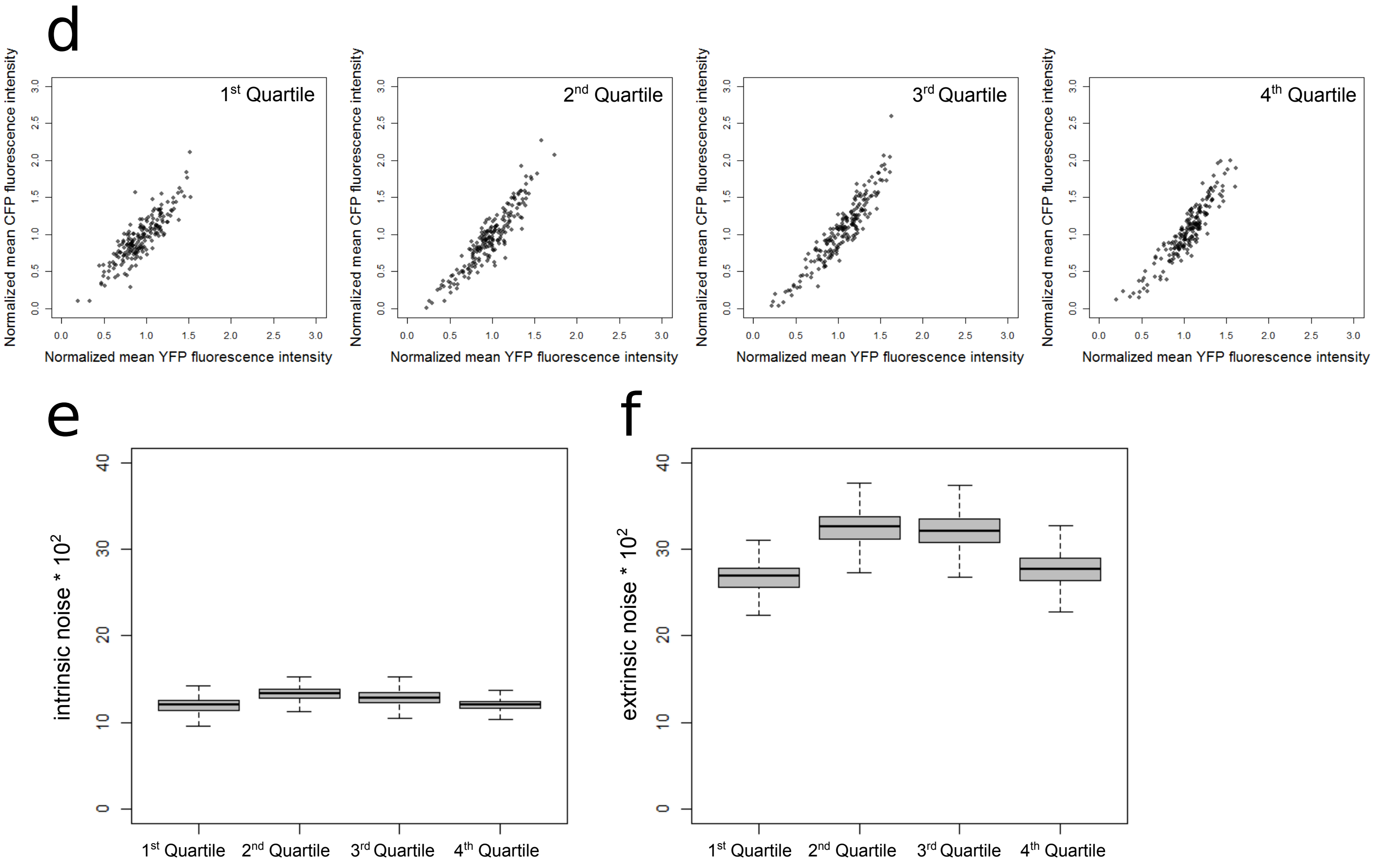

Supplementary Fig. 19: Analysis of noise with respect to the nucleus size of pavement cells of *p35S:2xNLS-YFP p35S:2xNLS-CFP* and *pUBQ10:2xNLS-YFP pUBQ10:2xNLS-CFP* plants. (A) Scatter plots of normalized mean CFP and YFP intensities of *p35S:2xNLS-YFP p35S:2x NLS-CFP* nuclei. Cells were separated into quartiles depended on their nucleus size and scatter plots are shown for each quartile. (B) Box plot analysis of intrinsic noise in *p35S:2xNLS-YFP p35S:2xNLS-CFP* plants (n=757 × 1000 cells) in four nuclear size quartiles. Intrinsic noise was similar in all four quartiles. (C) Box plot analysis of extrinsic noise in *p35S:2xNLS-YFP p35S:2xNLS-CFP* plants (n=757 × 1000) in four nuclear size quartiles. Extrinsic noise was higher in larger nuclei (3rd and 4th quartile) as compared to the 1st quartile (Bootstrap analysis; $p < 0.001$, Wilcoxon rank-sum test). (D) Scatter plot of normalized mean CFP and YFP intensities of *pUBQ10:2xNLS-YFP pUBQ10:2x NLS-CFP* nuclei for each quartile. (E) Box plot analysis of intrinsic noise of *pUBQ10:2xNLS-YFP pUBQ10:2xNLS-CFP* plants (n=775 × 1000 cells) in four nuclear size quartiles. Intrinsic noise was similar in all four quartiles. (F) Box plot analysis of extrinsic noise of *pUBQ10:2xNLS-YFP pUBQ10:2xNLS-CFP* plants (n=775 × 1000 cells) in four nuclear size quartiles. Extrinsic noise was higher in larger nuclei (2nd, 3rd and 4th quartile) as compared to the 1st quartile (Bootstrap analysis; $p < 0.001$, Wilcoxon rank-sum test).

Supplementary Fig. 20

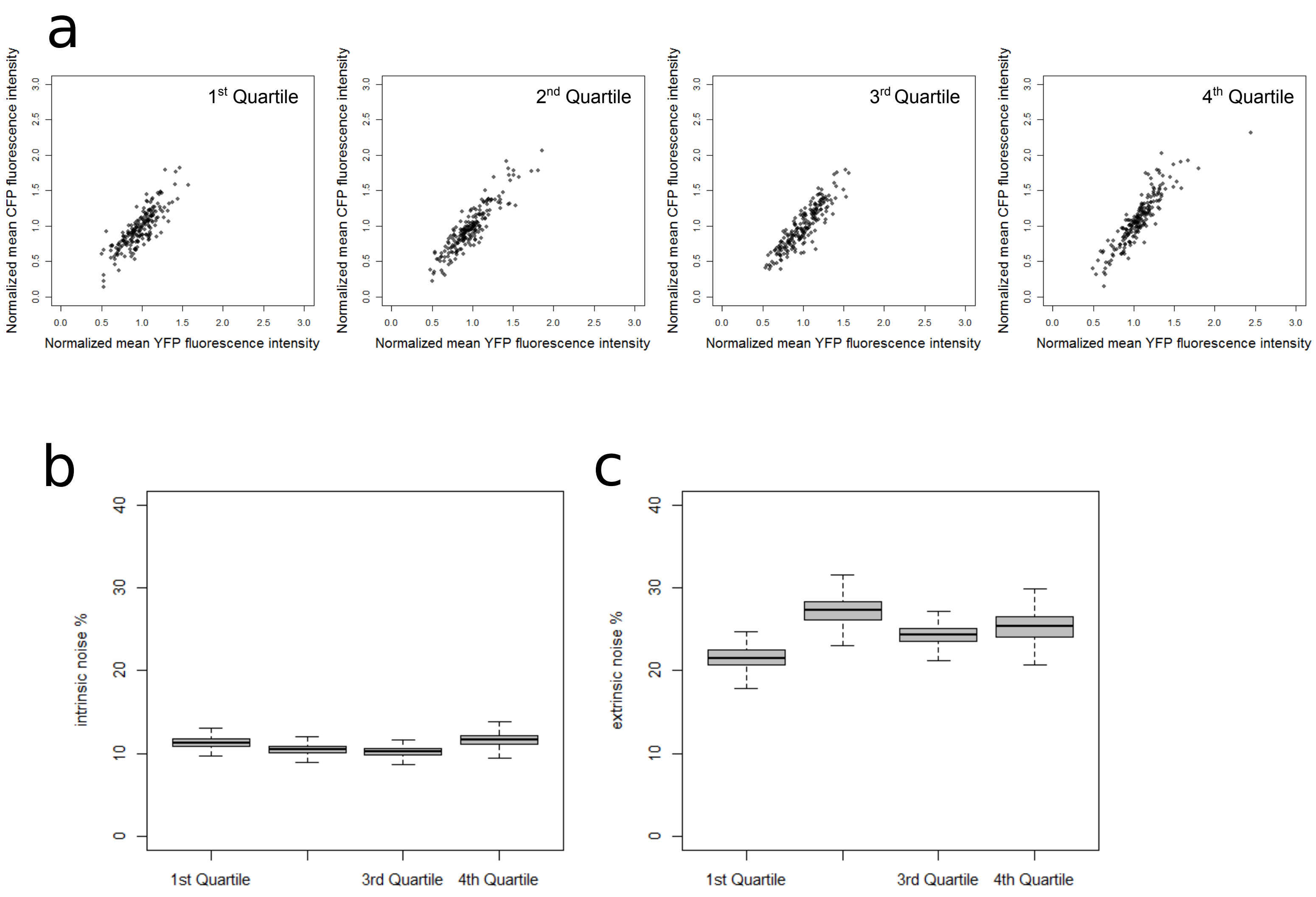

Supplementary Fig. 20: Analysis of noise with respect to the nucleus size of pavement cells of a second independently transformed *p35S:2xNLS-YFP p35S:2xNLS-CFP* line (Transgenic line 2).

(A) Scatter plot of normalized mean CFP and YFP intensities of *p35S:2xNLS-YFP p35S:2xNLS-CFP* nuclei for each quartile. (B) Box plot analysis of intrinsic noise of *p35S:2xNLS- YFP p35S:2xNLS-CFP* plants (n=796×1000 cells) in four nuclear size quartiles. Intrinsic noise was similar in all four quartiles. (C) Box plot analysis of extrinsic noise of *p35S:2xNLS-YFP p35S:2xNLS-CFP* plants (n=796 × 1000 cells) in four nuclear size quartiles. Extrinsic noise was higher in larger nuclei (2nd, 3rd and 4th quartile) as compared to the 1st quartile (Bootstrap analysis; $p < 0.001$, Wilcoxon rank-sum test)

Supplementary Fig. 21

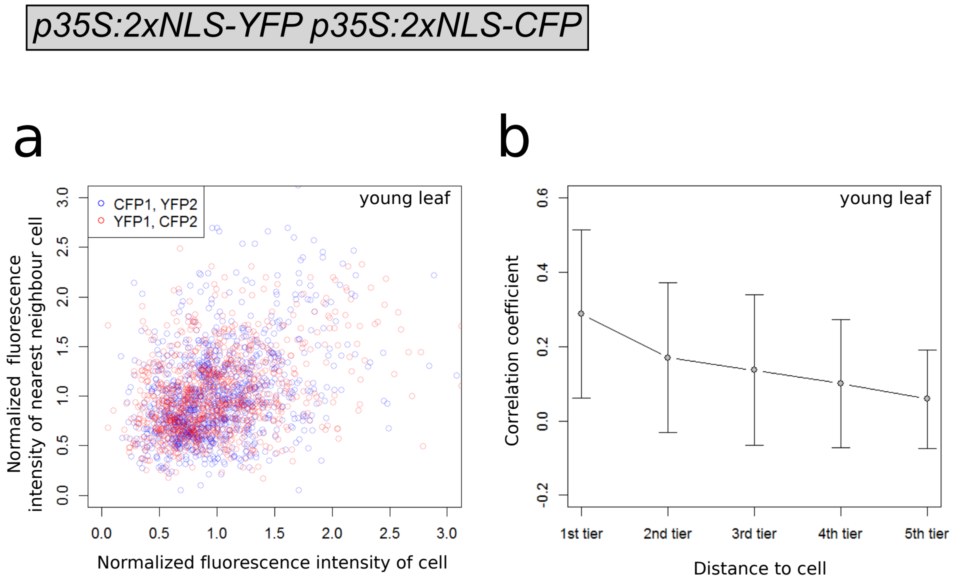

Supplementary Fig. 21: Nearest neighbour analysis of a second independently transformed *p35S:2xNLS-YFP p35S:2xNLS-CFP* line (Transgenic line 2).

(A) Scatter plot of *p35S:2xNLS-YFP p35S:2xNLS-CFP* young rosette leaves showing the normalized fluorescence intensities of cells plotted against the normalized fluorescence intensity of the nearest neighbour of the considered cells (neighbour cell with the lowest distance). Blue circles indicate the CFP fluorescence intensity of a cell (CFP1) plotted against the YFP fluorescence intensity of the nearest neighbouring cell (YFP2). Red circles show the YFP fluorescence intensity of a cell (YFP1) plotted against the CFP fluorescence intensity of the nearest neighbouring cell (CFP2) (n=1020 cells, r=0.423, p=0.0014, randomization test). (B) Dependency of the distance to the neighbouring cell and co-fluctuation in young rosette leaves of *p35S:2xNLS-YFP p35S:2xNLS-CFP*. Neighbouring cells were grouped into five tiers dependent on their distance (cell diameters) to the considered cell. Mean values and standard deviations are shown (n=39780 neighbourhood analyses). (C) Scatter plot of *p35S:2xNLS-YFP p35S:2xNLS-CFP* mature rosette leaves showing the normalized fluorescence intensities of cells plotted against the normalized fluorescence intensity of the nearest neighbour (n=796 cells, r = 0.014, p = 0.451, randomization test).

Supplementary Fig. 22

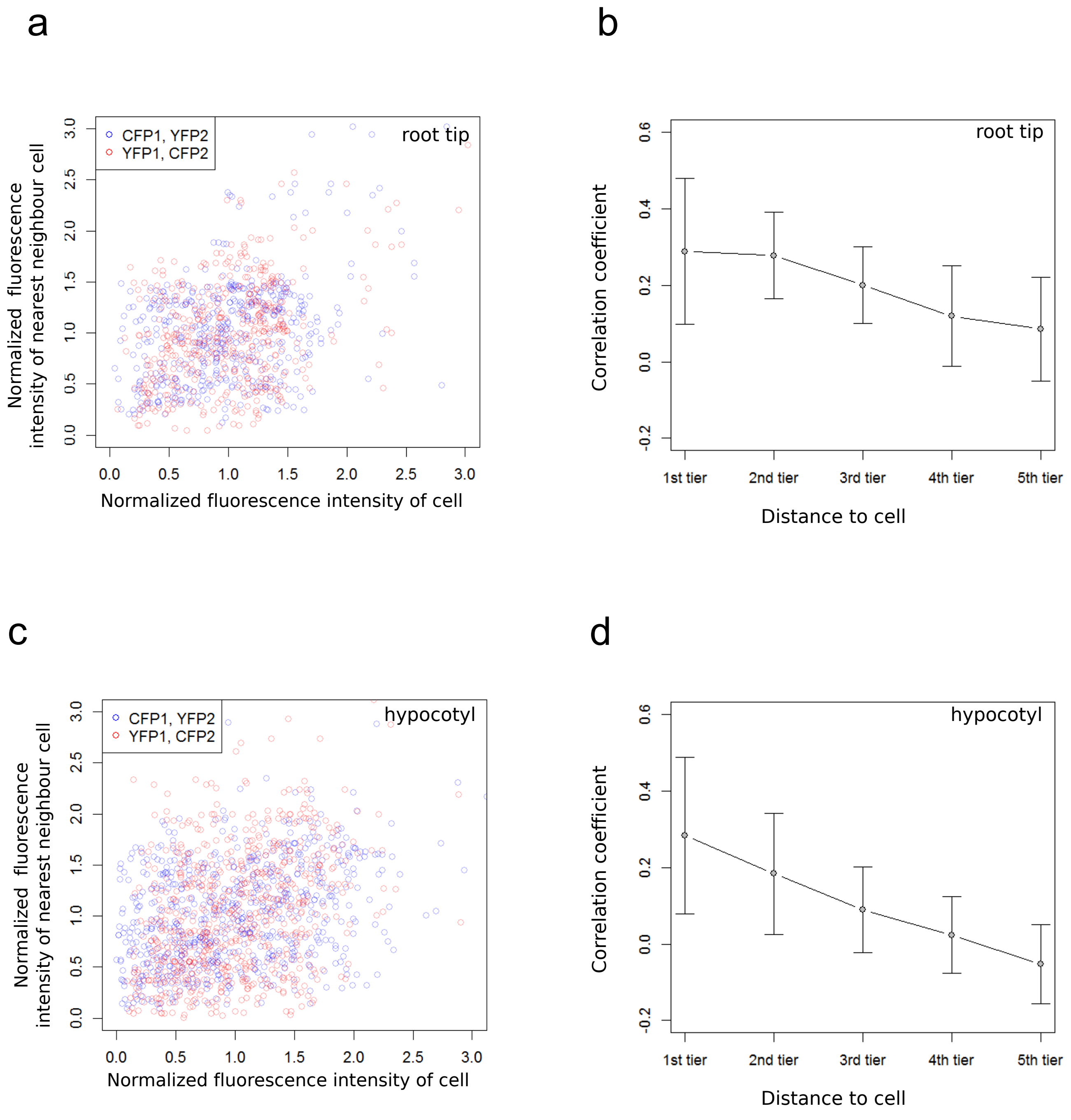

Supplementary Fig. 22: Nearest neighbour analysis of root tips and hypocotyls of *p35S:2xNLS-YFP p35S:2xNLS-CFP* line.

(A) Scatter plot of *p35S:2xNLS-YFP p35S:2xNLS-CFP* root tip cells showing the normalized fluorescence intensities of cells plotted against the normalized fluorescence intensity of the nearest neighbour of the considered cells (neighbour cell with the lowest distance). Blue circles indicate the CFP fluorescence intensity of a cell (CFP1) plotted against the YFP fluorescence intensity of the nearest neighbouring cell (YFP2). Red circles show the YFP fluorescence intensity of a cell (YFP1) plotted against the CFP fluorescence intensity of the nearest neighbouring cell (CFP2) (n=463 cells, r=0.447, p = 0.0008, randomization test). (B) Dependency of the distance to the neighbouring cell and co-fluctuation in root tip cells of *p35S:2xNLS-YFP p35S:2xNLS-CFP*. Neighbouring cells were grouped into five tiers dependent on their distance (cell diameters) to the considered cell. Mean values and standard deviations are shown (n=18057 (463 cells × 39 neighbouring cells) neighbourhood analyses). (C) Scatter plot of *p35S:2xNLS- YFP p35S:2xNLS-CFP* hypocotyl cells showing the normalized fluorescence intensities of cells plotted against the normalized fluorescence intensity of the nearest neighbour (n=690 cells; r = 0.379, p = 0.0, randomization test). (D) Dependency of the distance to the neighbouring cell and co-fluctuation in hypocotyl cells of *p35S:2xNLS-YFP p35S:2xNLS-CFP*. Neighbouring cells were grouped into five tiers dependent on their distance (cell diameters) to the considered cell. Mean values and standard deviations are shown (n=26910 (690 cells × 39 neighbouring cells) neighbourhood analyses).

Supplementary Fig. 23

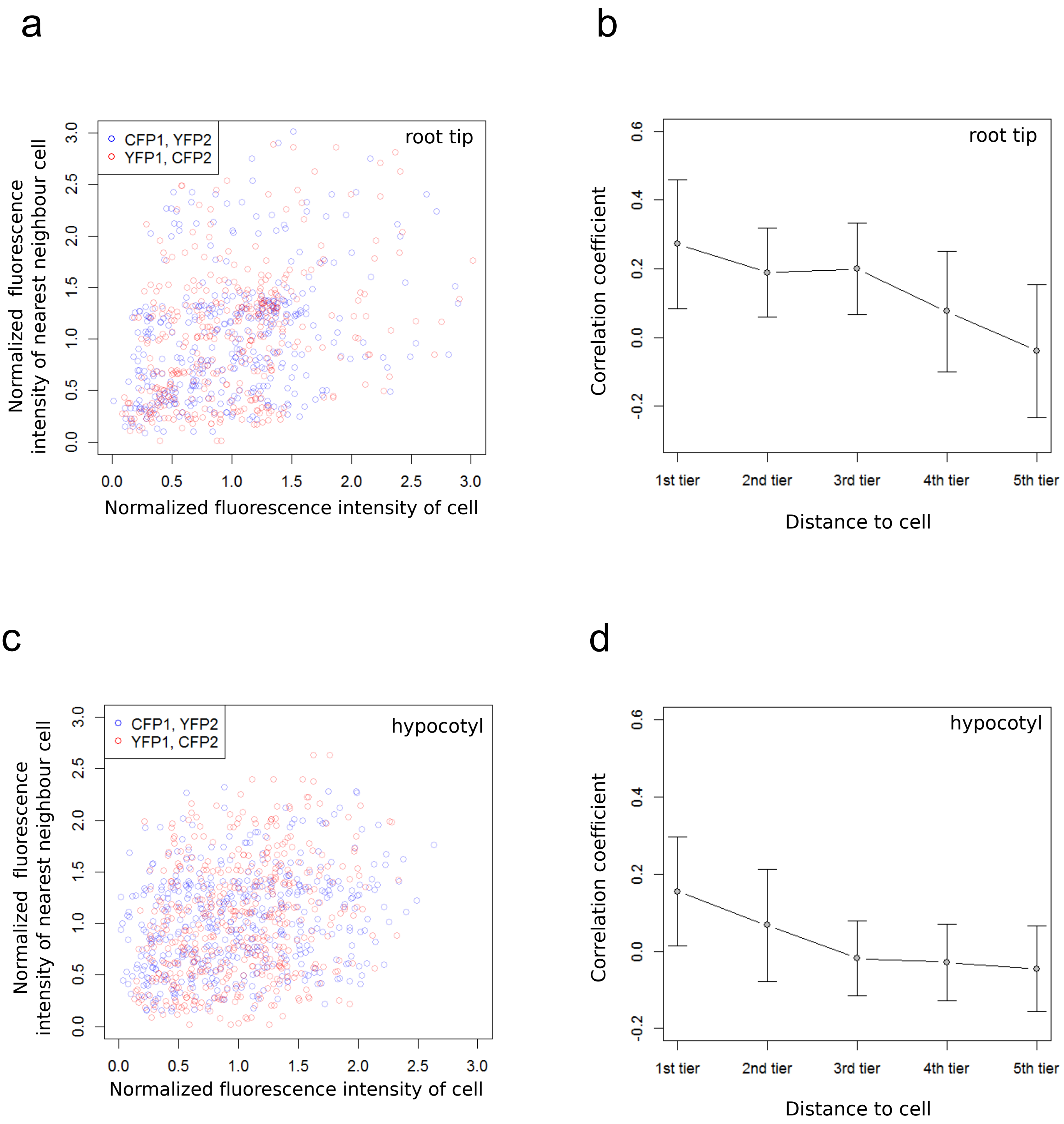

Supplementary Fig. 23: Nearest neighbour analysis of root tips and hypocotyls of a second independently transformed *p35S:2xNLS-YFP p35S:2xNLS-CFP* line (Transgenic line 2).

(A) Scatter plot of *p35S:2xNLS-YFP p35S:2xNLS-CFP* root tip cells showing the normalized fluorescence intensities of cells plotted against the normalized fluorescence intensity of the nearest neighbour of the considered cells (neighbour cell with the lowest distance). Blue circles indicate the CFP fluorescence intensity of a cell (CFP1) plotted against the YFP fluorescence intensity of the nearest neighbouring cell (YFP2). Red circles show the YFP fluorescence intensity of a cell (YFP1) plotted against the CFP fluorescence intensity of the nearest neighbouring cell (CFP2) (n=385 cells, r = 0.375, p = 0.0018, randomization test). (B) Dependency of the distance to the neighbouring cell and co-fluctuation in root tip cells of *p35S:2xNLS-YFP p35S:2xNLS-CFP*. Neighbouring cells were grouped into five tiers dependent on their distance (cell diameters) to the considered cell. Mean values and standard deviations are shown (n=15015 (385 cells × 39 neighbouring cells) neighbourhood analyses). (C) Scatter plot of *p35S:2xNLS-YFP p35S:2xNLS-CFP* hypocotyl cells showing the normalized fluorescence intensities of cells plotted against the normalized fluorescence intensity of the nearest neighbour (n=481 cells; r = 0.241, p = 0.0135, randomization test). (D) Dependency of the distance to the neighbouring cell and co-fluctuation in hypocotyl cells of *p35S:2xNLS-YFP p35S:2xNLS-CFP*. Neighbouring cells were grouped into five tiers dependent on their distance (cell diameters) to the considered cell. Mean values and standard deviations are shown (n=18759 (481 cells × 39 neighbouring cells) neighbourhood analyses).

Supplementary Fig. 24

a

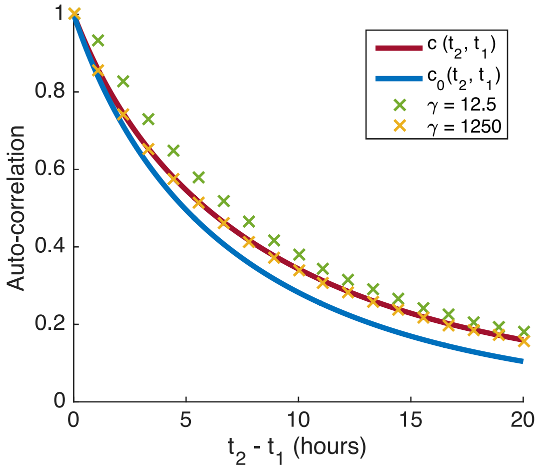

b

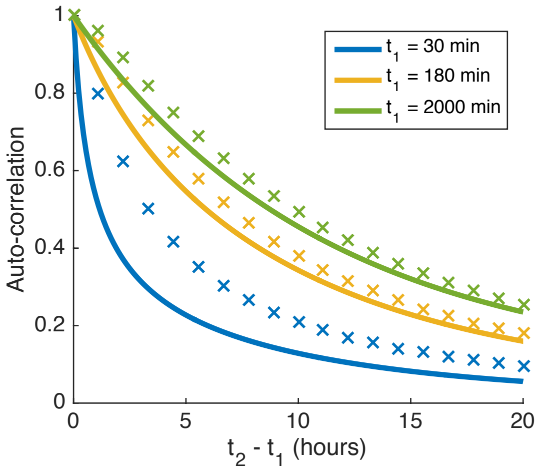

c

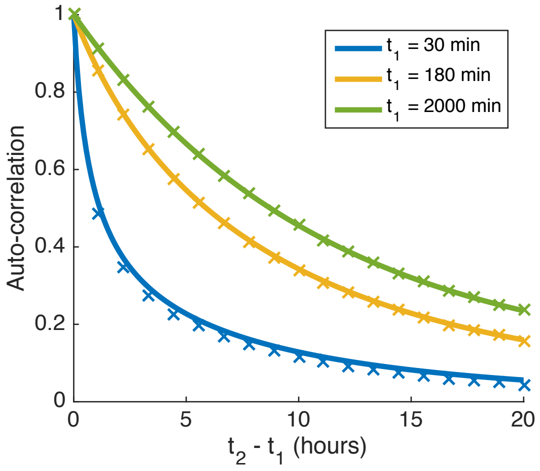

Supplementary Fig. 24: Simulation of the KikGR experiments.

(A, B) Auto-correlation of the reporter system computed for different t_1. Solid lines denote theoretical predictions of the auto-correlation given by Eq. (2), crosses denote the stochastic simulation results, computed from 10^5 trajectories. (B) Auto-correlation with $\gamma = 12.5$. (C) Auto-correlation with $\gamma = 1250$. Parameters are for (A - C): $\nu_0 = 2.25$, $d_1 = 0.09$. For $\gamma = 12.5$: $\langle \nu_1 \rangle = 45$, $\mathrm{Var}(\nu_1) = 8.1$, $d_0 = 1.125$ and for $\gamma = 1250$: $\langle \nu_1 \rangle = 4500$, $\mathrm{Var}(\nu_1) = 8.1 \times 10^4$, $d_0 = 112.5$.

Supplementary Fig. 25

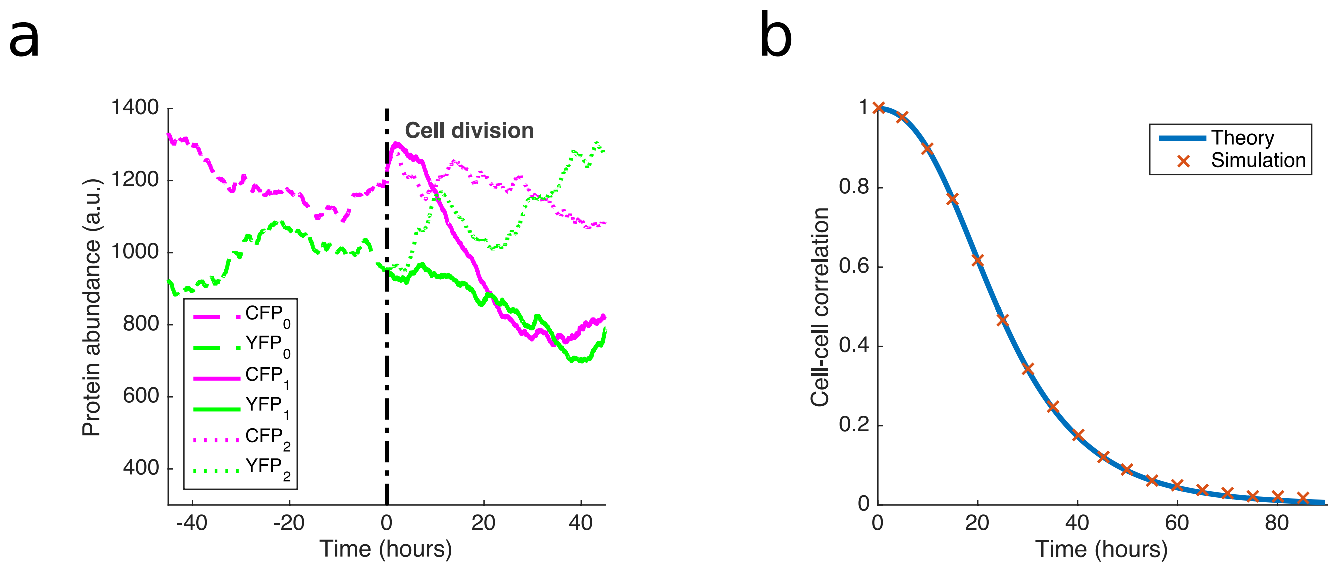

Supplementary Fig. 25: Correlation due to inheritance.

Cell division with dual reporter system. At $t = 0$ an identical copy of the mother cell is produced. All parameters of the two-stage model are inherited, besides the translational rates, which are in general different between mother and daughter cells. (A) Example trajectories of the situation before and after cell division are shown. The daughter cells inherit mRNA and protein content from the mother cell. All parameters of the two- stage gene expression model are as well inherited besides the translational rates, which are different between mother ($\nu_1 = 14.581$) and daughter cells ($\nu_1 = 10.449$ and $\nu_1 = 15.917$, respectively). The other parameters are: $\nu_0 = 2.25$, $d_0 = 1.125$, $d_1 = 0.029$. (B) Correlation calculated using Eq. (7) between daughter cells, computed from 10^5 simulated trajectories. The solid line denotes theoretical predictions of the auto-correlation given by Eq. (27), crosses denote the stochastic simulation results. Parameters are: $\nu_0 = 2.25$, $\langle \nu_1 \rangle = 14.5$, $\mathrm{Var}(\nu_1) = 5$, $d_0 = 1.125$, $d_1 = 0.029$.

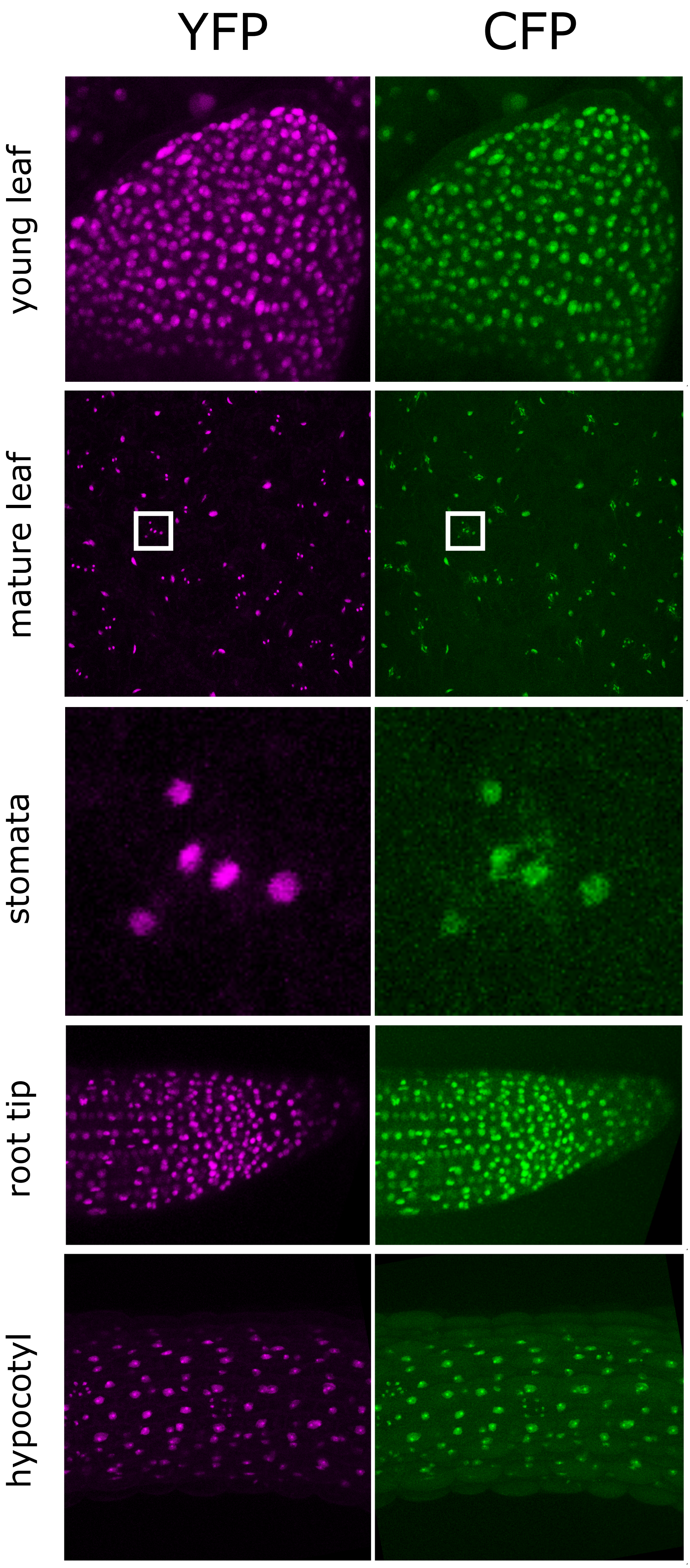

YFP
CFP
young leaf
mature leaf
stomata
root tip
hypocotyl

Supplementary Fig. 26: Raw images of different cell types.

Raw images are shown for the YFP and CFP channel for all cell types/tissues. The mean fluorescence intensities were in the following ranges (YFP, CFP). For Line 1: hypocotyl 110, 101, mature leaf 155, 118, root tip 112, 110, stomata 99, 106, young leaf 60, 83. For Line 2: hypocotyl 75, 90, mature leaf 114, 96, root tip 118, 116, stomata 134, 126, young leaf 39, 74. For the ubiquitin promoter line: mature leaf 141, 83, young leaf 58, 52.
